# The CDK inhibitor p57^Kip2^ enhances the activity of the transcriptional coactivator FHL2

**DOI:** 10.1038/s41598-020-62641-4

**Published:** 2020-04-28

**Authors:** Michael Keith Kullmann, Silvio Roland Podmirseg, Martina Roilo, Ludger Hengst

**Affiliations:** Institute of Medical Biochemistry, Biocenter, Medical University of Innsbruck, Innrain 80-82, A-6020 Innsbruck, Austria

**Keywords:** Oncogenes, Transcription

## Abstract

The eukaryotic cell cycle is negatively regulated by cyclin-dependent kinase inhibitors (CKIs). p57^**Kip2**^ is a member of the Cip/Kip family of CKIs and frequently inactivated by genomic mutations associated with human overgrowth disorders. There is increasing evidence for p57 to control cellular processes in addition to cell cycle and CDK regulation including transcription, apoptosis, migration or development. In order to obtain molecular insights to unknown functions of p57, we performed a protein interaction screen. We identified the transcription regulator four-and-a-half LIM-only protein 2 (FHL2) as a novel p57-binding protein. Co-immunoprecipitation and reporter gene assays were used to elucidate the physiological and functional relevance of p57/FHL2 interaction. We found in cancer cells that endogenous p57 and FHL2 are in a complex. We observed a substantial induction of established FHL2-regulated gene promoters by p57 in reporter gene experiments and detected strong induction of the intrinsic transactivation activity of FHL2. Treatment of cells with histone deacetylase (HDAC) inhibitors and binding of exogenous FHL2 to HDACs indicated repression of FHL2 transcription activity by HDACs. In the presence of the HDAC inhibitor sodium butyrate activation of FHL2 by p57 is abrogated suggesting that p57 shares a common pathway with HDAC inhibitors. p57 competes with HDACs for FHL2 binding which might partly explain the mechanism of FHL2 activation by p57. These results suggest a novel function of p57 in transcription regulation.

## Introduction

Cell proliferation is negatively regulated by proteins of the Cip/Kip-family^1^. Using a conserved N-terminal cyclin/CDK binding motif, the three family members p21^Cip1/Waf1^ (p21), p27^Kip1^ (p27) and p57^Kip2^ (p57) regulate the activity of cyclin/CDK complexes. In their unmodified form, binding inhibits the kinase activity of the associated cyclin/CDK complex. Cip/Kip proteins were reported as candidate tumor suppressor genes^2^. In contrast to mice deficient for p21 or p27, p57 knock-out mice show severe developmental defects. The observed prenatal lethality resembles many features of Beckwith-Wiedemann syndrome (BWS), a genetic disorder characterised by developmental defects and predisposition to the development of tumors in humans^3,4^. Interestingly, alterations of the p57 coding gene *CDKN1C* have been frequently observed in BWS patients and genetic and epigenetic alterations impairing p57 expression or function are the most frequent cause of BWS^5–7^. However, some BWS patients carry mutations outside the cyclin/CDK binding domain and mouse knock-in studies revealed a CDK-independent contribution of p57 in BWS^8^. Therefore, not all of the observed phenotypes can be attributed to the ability of p57 to bind and to inhibit cyclin/CDK complexes^8,9^. Some phenotypes of p57-deficient mice were even enhanced when a cyclin/CDK binding deficient mutant (p57^CK−^) was expressed in mice, indicating additional dominant effects of the p57^CK−^ mutant by so far unknown mechanisms^8^. Several recent publications highlighted a role of the closely related p27 protein as a transcription regulator which can be CDK-dependent and CDK-independent^10–13^. p57 has also been reported to directly and indirectly regulate transcription; it binds and inactivates CDK7 and CDK9 and interacts with the transcription factor E2F1 thereby repressing E2F1 regulated genes^14^. In the proposed model p57 is recruited to promoter sites by E2F1 where it can bind CDK7 or CDK9 and inhibit the phosphorylation of RNA Polymerase II C-terminal repeat domain (CTD)^14^. Transcriptional regulation by p57 was also described to play a role in myogenesis and neurogenesis^15,16^. p57 stabilises the transcription factor myoD by direct binding or by inhibiting CDK2 and thereby promoting myogenesis in a cell culture model^15,17^. In addition, p57 was reported to repress neuronal differentiation after mitogen withdrawal and suggested to play a role as a context-dependent repressor of neurogenic transcription factors like Mash1, NeuroD and Nex/Math2^16^.

In order to gain more insight into novel functions of p57, we aimed to identify novel p57 binding partners. Therefore, we performed a yeast two-hybrid screen and obtained the protein FHL2 as a novel p57-interactor.

FHL2 is a multifunctional LIM domain only protein which binds cellular proteins via its LIM domains and thereby regulates various cellular processes^18^. Although FHL2 does not directly bind to DNA, it modulates the activity of several transcription factors^19,20^. FHL2 was first described to bind to the hormone-activated androgen receptor (AR) which increases the activity of AR-dependent reporter genes^21^. FHL2 is expressed in the cytoplasm and the nucleus. Interestingly, in several cancer types high levels of nuclear FHL2 correlate with disease progression towards a malignant state. This indicates that FHL2 dependent transcription contributes to cancer development and progression^22,23^.

Here we report that p57 strongly activates FHL2 transactivation function and induces the activity of known FHL2-regulated promoters. We provide experimental evidence supporting the hypothesis that FHL2 is repressed by HDACs and p57 relieves this repression by competing with HDACs for FHL2-binding. FHL2 and p57 might regulate transcription as components of chromatin remodeling complexes.

## Materials and Methods

### Plasmids and oligonucleotide sequences

Detailed descriptions of novel plasmid constructs, including cloning strategies and sequences of oligonucleotides used are presented in Supplementary information.

### Cell culture, transfections and cell lysis

The human embryonic kidney (HEK) cell lines 293 and 293 T, the human cervix carcinoma cell line HeLa and the colon carcinoma cell line HRT-18 (also termed HCT-8) were cultured in DMEM (Sigma-Aldrich, St. Louis, MO, USA) supplemented with 10% FBS (PAA) plus 100 U/ml penicillin, 100 µg/ml streptomycin (Sigma-Aldrich, St. Louis, MO, USA) according to ATCC guidelines. Cells were treated with 1 nM of the synthetic androgen R1881 (Organon) as described^24^. The histone deacetylase inhibitors sodium butyrate (NaBu) and Trichostatin A (TSA) were both purchased from Sigma-Aldrich, St. Louis, MO, USA and used from 1 and 0.66 M stock solutions, dissolved in water (NaBu) or DMSO (TSA). 293 and 293 T cells were transfected by calcium phosphate precipitation^25^, HeLa cells by Lipofectamine 2000 (Thermo Fisher Scientific). Cells were lysed in Laemmli buffer^26^ or IP-buffer (50 mM Tris pH 7.5, 150 mM NaCl, 0.5% NP-40 and protease inhibitor cocktail (Sigma Aldrich, St Louis, MO, USA) using an ultrasonic homogeniser (Sonoplus, Bandelin, Berlin, Germany)^27^.

### Subcellular fractionation

Crude cytoplasmic and nuclear fractions from HRT-18 cells for subsequent use in immunoprecipitation experiments were obtained by using digitonin as a detergent^28^. In order to avoid cytoplasm-contaminated nuclear fractions and to minimize loss of nuclear proteins during the procedure minor modifications of the protocol were applied. All steps were done at 4 °C. Briefly, 10 ×15 cm cell culture plates of subconfluent HRT-18 cells were rinsed twice with PBS and carefully scraped in 10 ml PBS per plate and collected into 50 ml Falcon tubes. After centrifugation (300 g for 5 minutes), cell pellets were washed again with 10 ml PBS and transferred to a 15 ml Falcon tube. After another centrifugation step supernatant was carefully removed and cell pellet volume judged. Cells were resuspended in 3x pellet volume digitonin buffer (0.3 mg/ml digitonin, 50 mM Tris pH 7.5, 150 mM NaCl and protease inhibitor cocktail (Sigma Aldrich, St Louis, MO, USA)) and immediately centrifuged at 1000 g for 3 min. Before, a small aliquot was tested for trypanblue exclusion and only when more than 90% of cells were “blue” fractionation was carried on. Supernatant was collected as cytoplasmic fraction. The nuclear pellet was washed once with 5 pellet volumes 50 mM Tris pH 7.5, 150 mM NaCl and extracted in 3 pellet volumes IP-buffer (50 mM Tris pH 7.5, 150 mM NaCl, 0.5% IGEPAL CA-630 and protease inhibitor cocktail (Sigma Aldrich, St Louis, MO, USA)) using an ultrasonic homogeniser (Sonoplus, Bandelin, Berlin, Germany). The nuclear extract was cleared by centrifugation at 25000 g for 30 min. and the supernatant saved as nuclear fraction. Similar volumes (typically 1 ml) of cytoplasmic and nuclear fractions were used in immunoprecipitation experiments.

### Yeast two-hybrid assay and interaction experiments in yeast

The “MATCHMAKER Two-hybrid System” (Clontech) was applied with minor modifications. The plasmid pAS2-1-p57 was stably integrated into the *S. cerevisiae* PJ69-4A strain to obtain the “bait” strain PJ69-4A-p57. The “Human HeLa MATCHMAKER cDNA library” (HL4048AH, Clontech) was used to transform PJ69-4A-p57 with “prey” expressing cDNAs in the vector pACT2. Isolated pACT2 plasmids were verified by retransformation. Finally, the plasmids from 19 clones were recovered and sequenced. Eight plasmids contained the coding sequence of FHL2 in frame with the Gal4 transactivation domain. Specific interaction with FHL2 was tested by transforming a PJ69-4A strain containing stably integrated pACT2-FHL2 plasmid together with pAS2-1-p57 or pAS2-1-p21 or pAS2-1-p27 or pAS2-1-RanGAP^(tail)^.

### Western blot and immunoprecipitation

For protein expression and IP experiments cells were harvested 24 to 30 hours after transfection and stored as cell pellets at −80 °C. Levels of the individual proteins in extracts were determined by Western blotting and if necessary adjusted before IP. Antibodies for IP were covalently coupled to protein A-agarose (Immunosorb A; Medicargo, Sweden). For HA- and FLAG-IPs antibody-coupled beads were incubated with cellular extracts for 4 hours and for IP of p57 over night at 4 °C under continous rotation. After incubation beads were washed five times with IP-buffer and proteins eluted from beads by incubation in Laemmli-buffer without DTT at 40 °C for 10 minutes and subjected to Western blotting. Primary antibodies were detected with horseradish peroxidase-coupled secondary antibodies and enhanced chemoluminescence (ECL).

Following antibodies were used for detection or IP of p57 and FHL2: rabbit polyclonal p57 (C-20): sc-1040, mouse monoclonal p57 (KP39): sc-56341, mouse monoclonal FHL-2 (F4B2-B11): sc-52667 (Santa Cruz, Santa Cruz, CA, USA), for detection of PSTAIR-motif containing CDKs: mouse monoclonal anti-PSTAIRE^29^, for FLAG-epitope tagged proteins: rabbit polyclonal ANTI-FLAG and mouse monoclonal ANTI-FLAG M2 (Sigma-Aldrich, St. Louis, MO, USA -Aldrich, St. Louis, MO, USA) and HA-epitope tagged proteins: mouse monoclonal anti-HA (12CA5 ab16918, Abcam, Cambridge, MA, USA). For subcellular fractionation rabbit polyclonal Lamin A/C: 10298-1-AP (Proteintech) and mouse monoclonal GAPDH: sc-47724 (Santa Cruz, Santa Cruz, CA, USA) were used.

### Luciferase reporter gene assays

The cell line 293FR was generated by transfecting 293 cells with pFR-Luc together with a puromycin-resistance conferring plasmid. Single colonies were isolated under puromycin selection (1 µg/mL) and tested for activation by pFA-c-fos (Stratagene, La Jolla, CA, USA). From several positive clones one was selected for low basal and considerable Gal-c-Fos induced reporter activity. MMTV-promoter experiments were performed with 293 cells in 24-well format. Typically, 250 ng MMTV-Luc, 12.5 ng pSG5-hAR, 40 ng pDEST-3xHA-p57 and 2 ng Ubi-RLuc were adjusted to 2 µg plasmid DNA with pUC18 and transfected. Four hours after transfection cells were treated with R1881 (10^−9^ M) for 24 hours. Cyclin D1- and MMP1-promoter experiments were performed with HeLa cells in 12-well format. Typically, 1.5 µg plasmid DNA was composed of 0.3 µg reporter plasmids cyclin D1-Luc or MMP1-Luc or pGL3-Basic together with 1.2 µg pDEST-3xHA-p57 and 10 ng Ubi-RLuc. Gal4-reporter experiments were performed with 293FR-cells in 6-well format. Typically, a total of 5 µg DNA was composed of 2 µg Gal-fusion expressing plasmids pSG424-FHL2 or pSG424-FHL2-LIM1/2-2, 3 µg pDEST-3xHA-p57 or 3 µg pDEST-3xHA-p27 and 20 ng UbiRluc. In RhoA experiments 1.5 µg pDEST-3xHA-p57 were co-transfected with 1.5 µg pEF-neo or pEF-RhoAV14 or pEF-RhoAN17. pDEST-Control vector was used as the non-expressing reference. Cells were lysed and luciferase activities determined using the Dual-Luciferase Assay system (Promega, Madison, WI, USA) and a Lumat LB9501 luminometer (Berthold). For quantification firefly luciferase counts were divided by Renilla luciferase counts and expressed relative to control transfected as “fold activation”.

### Cell cycle analysis

Cell cycle phase distributions of transfected cells were done as described earlier^30^. Briefly, 293 T cells were transfected with 100 ng pBB14, 30 ng pDEST-3xFLAG-p57 and 200 ng pDEST-3xFLAG-FHL2 or pDEST-Control. Rc/CMV cyclin E was transfected as a positive control instead of pDEST-3xFLAG-FHL2. Forty hours after transfection cells were trypsinised and fixed in 70% ice-cold ethanol and stored at −20 °C. The expression of the integral membrane fusion protein Us9-gfp from pBB14 is resistant to ethanol-fixation and green fluorescence is therefore quantitatively maintained in cells. DNA of fixed cells was stained using propidium-iodide and cells subjected to FACS analysis. Doublets of cells were excluded by FL2 (PI)-width vs FL2 (PI)-area dot blots, GFP-positive (transfected) cells were gated in FL2 (PI) vs FL1 (GFP) dot blots. Cell cycle distribution was analysed in FHL2 (PI) histograms with a FACScan flow cytometer (BD Bioscience) and quantified with FlowJo software (Tree Star). The cell cycle distribution from control (no p57, GFP-positive) transfected cells is not considerably different to untransfected (GFP-negative) cells.

### Immunofluorescence and fluorescence microscopy

For immunofluorescence HeLa cells were seeded on glass cover slips and grown in 12-well plates. Cells were transfected with FLAG-FHL2 and/or HA-p57-wt encoding plasmids using PEI (polyethylenimine) as transfection reagent^31^. After 30 hours cells were fixed using 4% paraformaldehyde and permeabilised with 0.1% Triton X-100 (#3051, Roth). After blocking with 0.5% gelatin (G1890, Sigma-Aldrich), samples were incubated with the following primary antibodies: rabbit polyclonal p57 ((C20) sc-1040, Santa Cruz) and monoclonal mouse anti-FLAG (M2 #F3165, Sigma-Aldrich). After washing, cells were incubated with fluorescence labeled antibodies 488 and 555 (Alexa Fluor, Invitrogen). Cells were mounted in a DAPI containing mounting medium (ab104139, Abcam). Analysis was performed on a Leica DMi8 inverted widefield microscope.

For fluorescence microscopy HeLa cells were grown in chambered coverslips (#80826, IBIDI, Planegg, Bavaria, Germany). Cells were transfected with Cherry-FHL2 and/or YFP-p57-wt or p57-Nt/p57-Nt-NLS1 encoding plasmids using PEI or Polyfect as transfection reagent. YFP (yellow fluorescent protein) was excited at 510 nm and mCherry at 550 nm. Analysis was performed on a Leica DMi8 inverted widefield microscope.

### Size exclusion chromatography (SEC)

SEC of protein complexes was basically done as described before^32^. Briefly, 293 T cells were transfected with pCruz-OctA-FHL2 and pCruz-OctA-p57 and harvested 40 hours later. Extracts were loaded onto a prepacked Superose 6 column (GE Healthcare Life Sciences) in 150 mM NaCl, 50 mM Tris (pH 7.5), 1 mM PMSF, and 1 mM DTT. Size exclusion chromatography was performed by collecting fractions (0.5 ml) at a flow rate of 0.6 ml/min at 4 °C by isocratic elution over 1.5 column volumes using an FPLC/HPLC ÄKTA Purifier (GE Healthcare Life Sciences). Fractions were analysed using SDS-PAGE and Western blot detection. Molecular weight marker kit MWGF200 (Sigma Aldrich, St Louis, MO, USA) was used for molecular mass determination.

### Statistical analysis

Statistical significance was evaluated by the parametric Student’s unpaired two-tailed t test using GraphPad Prism version 8.1. Values of p < 0.05 were considered significant and p < 0.01 highly significant. The data are presented as mean ± standard deviation (SD).

## Results

### FHL2 binds to p57

In order to identify novel p57 interacting proteins we performed a yeast two-hybrid screen with p57 fused to the DNA binding domain (DBD) of Gal4 as a bait. The yeast strain PJ64-4A-p57 was transformed with a prey library of HeLa cDNAs fused to the Gal4 transactivation domain. Prey plasmids were isolated from 42 colonies, sequenced and interactions verified by retransformation of the plasmids into PJ64-4A-p57. Of these 42 clones, 8 contained the ORF of the transcriptional coactivator four-and-a-half-LIM only protein 2 (FHL2) in frame with the Gal4 DBD. In addition, three clones encoding the known p57 interacting proteins cyclin D1 (two clones) and cyclin D3 (one clone) confirmed specificity of the assay.

**Figure 1:**
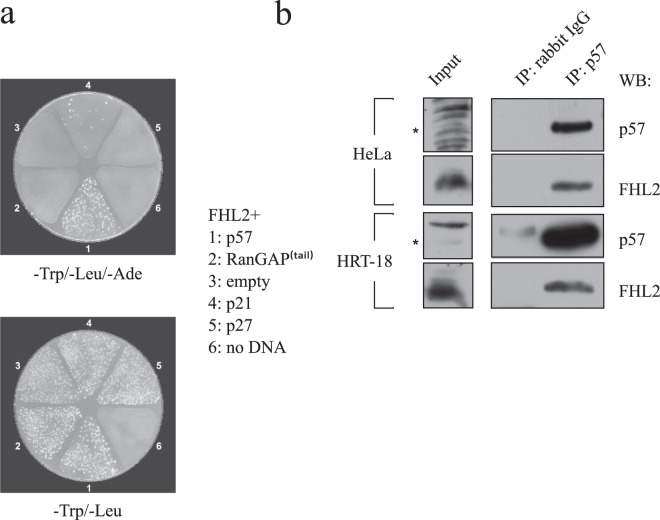
FHL2 interacts with p57. **(a)** Identification of FHL2 as a p57 interacting protein in a yeast two-hybrid screen. The lower panel represents growth of colonies on plates containing media lacking tryptophan and leucine (-Trp/-Leu) selecting for the presence of the bait and prey plasmids (“1–5”). The upper panel shows colonies grown following replica plating from -Trp/-Leu-plates onto plates containing selective media lacking tryptophan, leucine and adenine (-Trp/-Leu/-Ade). Colony growth (“1” / p57) indicates an interaction of expressed prey protein with the bait FHL2. RanGAP ^(tail)^ (“2”) served as a negative control. **(b)** Endogenous FHL2 and p57 form complexes in mammalian cells. Western blot analysis of cellular extracts derived from human cervix (HeLa) and colon carcinoma (HRT-18) cell lines after immunoprecipitation with a rabbit polyclonal p57-specific antibody. Coprecipitated FHL2 was detected with a mouse monoclonal FHL2-specific antibody. One percent of the protein amount used in IP was analysed on the same gel as input to control for the presence of p57 and FHL2. Asterisks left to the panels indicate p57 signals.

Due to the high degree of amino acid and structural conservation between the cyclin/CDK binding domains of the Cip/Kip proteins, we aimed to determine whether binding of FHL2 is specific for p57. Therefore, we performed interaction studies in yeast with FHL2 and the three human Cip/Kip proteins. We transformed PJ64-4A with pACT2-FHL2 together with bait plasmids expressing the yeast GAL4 DBD fused to full-length p57, p27 or p21 (pAS2-1-p57, pAS2-1-p27, pAS2-1-p21) or to the unrelated protein RanGAP (pAS2-1-RanGAP^(tail)^). Yeast colonies grew primarily in the presence of p57, indicating a specific interaction of p57 with FHL2 (Fig. 1a, upper panel). A few colonies could also be observed under conditions of p21 expression, which might indicate a weak interaction of p21 to FHL2.

We next aimed to confirm this interaction in mammalian cells. Both proteins are expressed in HeLa (cervix carcinoma) and HRT-18 (colon carcinoma) cells. Endogenous FHL2 coprecipitated with endogenous p57 from both cell lines (Fig. 1b), indicating that p57/FHL2 are in a stable complex *in vivo*.

### p57 binds to LIM-domains 2 to 4 of FHL2

**Figure 2:**
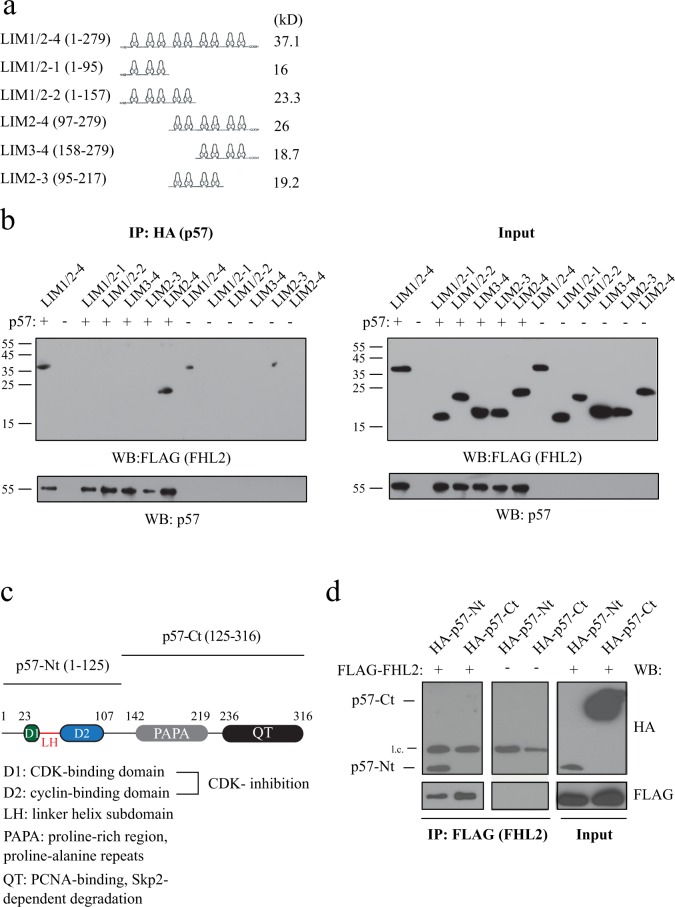
Mapping of interaction domains in FHL2 and p57. **(a)** Domain structure of FHL2 (LIM1/2–4) and the LIM domain deletion mutants. Each LIM domain is schematically represented by the two zinc finger domains. In brackets are the corresponding amino acids of FHL2 (1–279) or of the truncated proteins. The proteins were expressed with an N-terminal FLAG tag. The calculated molecular weight of the proteins is indicated. **(b)** p57 binds to LIM-domains 2–4 of FHL2. Full-length FLAG-FHL2 or the FLAG-tagged LIM domain deletion mutants were expressed (−) or co-expressed (+) with HA-p57 in 293 T cells. p57 was immunoprecipitated with anti-HA-antibodies. The immunoprecipitates were analysed in western blots and the bound FHL2 or LIM domain deletion mutants were detected with anti-FLAG-antibodies (left panel). One percent of protein extract used for IP served as input control (right panel). **(c)** Schematic representation of human p57 and the N-terminal and C-terminal fragments. Characteristic domains of p57 are indicated. Numbers indicate positions of amino acids flanking distinct domains. Amino acid including the N-terminal (p57-Nt) and C-terminal (p57-Ct) domains are shown in brackets. **(d)** FHL2 binds to the amino terminus of p57. HA-p57 amino terminal (p57-Nt) and carboxyterminal (p57-Ct) truncation mutants were expressed in 293 T cells together with FLAG-FHL2. FHL2 was immunoprecipitated from lysates using mouse anti-FLAG antibodies. Precipitated FHL2 was detected with rabbit anti-FLAG (left top panel) and coprecipitated p57 wildtype or mutants with anti-HA antibodies by Western blotting (left bottom panel). One percent of protein extract used for immunoprecipitation was loaded on the same gel and served as an input control (right panels). “l.c.” indicates signals originating from the light chain of the FLAG antibody.

FHL2 consists of four full and one half LIM-domains. To determine the structural requirements for p57 binding, we generated a series of deletion mutants of FLAG-tagged FHL2 (Fig. 2a). These mutants were co-transfected with HA-tagged p57 into 293 T cells. Efficient binding to p57 was only observed for full length FHL2 and the LIM2-4 mutant, whereas all other mutants, where the LIM-domains 2-4 were disrupted or absent, failed to stably bind to p57 (Fig. 2b, left panel). These results indicated that LIM-domains 2–4 are crucial for p57 binding.

### FHL2 binds to the amino-terminus of p57

Functional and structural domains of p57 have been characterised within the N- and the C-terminal part of the protein (Fig. 2c). To obtain more insight into the potential functional consequences of FHL2 binding, we mapped the required interaction domain of p57. Therefore, we transfected FHL2 together with either the N- (Nt) or the C-terminal (Ct) domains of p57 and tested their ability to bind to FHL2. The p57-Nt mutant consists of aa 1–125 and harbors the CDK-inhibitory domain with the regions binding to the cyclin and the CDK subunit. The p57-Ct mutant consists of aa 125 to 316 which includes the QT-domain with a phosphodegron important for ubiquitin-dependent degradation of p57 and the PCNA binding domain (Fig. 2c). Both mutants of p57 were expressed as HA-tagged proteins and co-expressed with FLAG-tagged FHL2. Using FLAG IPs, only the p57-Nt mutant coimmunoprecipitated with FHL2, whereas the larger and stronger expressed p57-Ct protein did not (Fig. 2d).

These data suggest that FHL2 binds to the N-terminal part of p57 which harbors domains which are essential and sufficient for cyclin/CDK binding and CDK inhibition. Therefore, FHL2 might compete with CDKs and cyclins for p57 binding and thereby interfere with the CDK inhibitory function of p57.

### p57 and FHL2 do not alter each other’s subcellular localization

**Figure 3:**
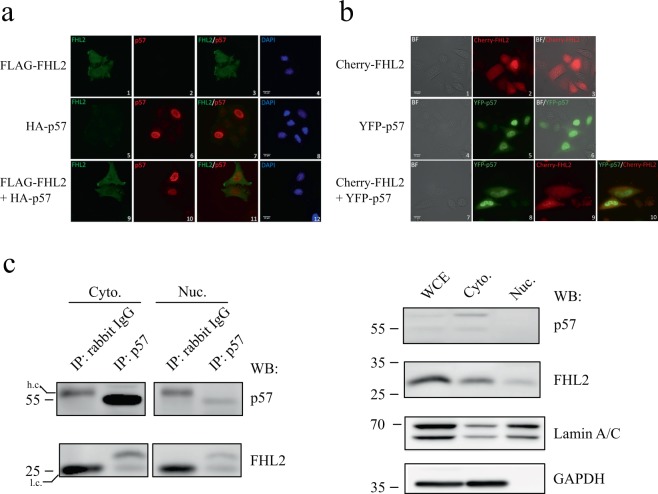
FHL2 and p57 co-localisation. **(a)** Analysis of FHL2 and p57 subcellular localization by immunofluorescence. FLAG-FHL2 and/or HA-p57 were overexpressed in HeLa cells as indicated. In fixed cells the localization of FHL2 was determined with FLAG-tag specific antibodies and the localization of p57 was determined with p57 specific antibodies and fluorescent labeled secondary antibodies. DNA was stained with DAPI. Scale bar represents 15 µm. **(b)** Localization of FHL2 and p57 in living cells. YFP-p57 and/or Cherry-FHL2 were overexpressed in HeLa cells. Proteins were detected by excitation of the fused fluorophores. Scale bar represents 15 µm. Bright field (BF). **(c)** Endogenous p57 and FHL2 are in a complex in the nucleus of HRT-18 cells. Crude subcellular fractionation of HRT-18 cells followed by immunoprecipitation of p57 and immunoblotting for co-precipitated FHL2. Left panel: Co-immunoprecipitation of FHL2 using rabbit polyclonal p57 antibodies compared to rabbit IgG control IP analysed by immunoblotting using p57- and FHL2-specific mouse monoclonal antibodies. Light and heavy chains (l.c./h.c.) are indicated. Right panel: Expression of FHL2 and p57 in whole cell extracts (WCE), cytoplasmic (Cyto.) and nuclear (Nuc.) fractions analysed by immunoblotting using p57- and FHL2-specific mouse monoclonal antibodies. Antibodies specific for GAPDH and Lamin A/C control for the purity of nuclear and cytoplasmic fractions.

FHL2 predominantly localises in the cytoplasm, whereas p57 is primarily expressed in the nucleus^21,33^. To characterise the interaction of FHL2 and p57, we investigated if exogenous co-expression of FHL2 and p57 might alter their subcellular distribution. The localisation of FHL2 and p57 in HeLa cells was determined by fluorescence microscopy in fixed as well as in living cells. As expected, p57 predominantly localises to the nucleus and to a small fraction in the cytoplasm (Fig. 3a, panels 6, 7 and 10; Fig. 3b, panels 5, 6 and 8). FHL2 is mainly localised in the cytoplasm, particularly in the periphery and at focal adhesion sites (Fig. 3a, panels 1, 3 and 9; Fig. 3b, panels 2, 3 and 9). However, the protein is also present in the nucleus. Importantly, co-expression of the two proteins did not alter their subcellular localization; it did neither increase cytoplasmic localization of p57 (Fig. 3a, compare panels 6 and 10; Fig. 3b, compare panels 5 and 8) nor alter the subcellular localization of FHL2 (Fig. 3a, compare panels 1 and 9; Fig. 3b, compare panels 2 and 9). This suggests that a functional interaction of p57 and FHL2 might occur in the nucleus. To validate this assumption we performed co-immunoprecipitation experiments upon subcellular fractionation of HRT-18 cells. We could detect FHL2 in the nuclear fraction after immunoprecipitation of p57 (Fig. 3c) further indicating a functional interaction of p57 and FHL2 in the nucleus.

### FHL2 does not reverse p57 induced cell cycle arrest

**Figure 4:**
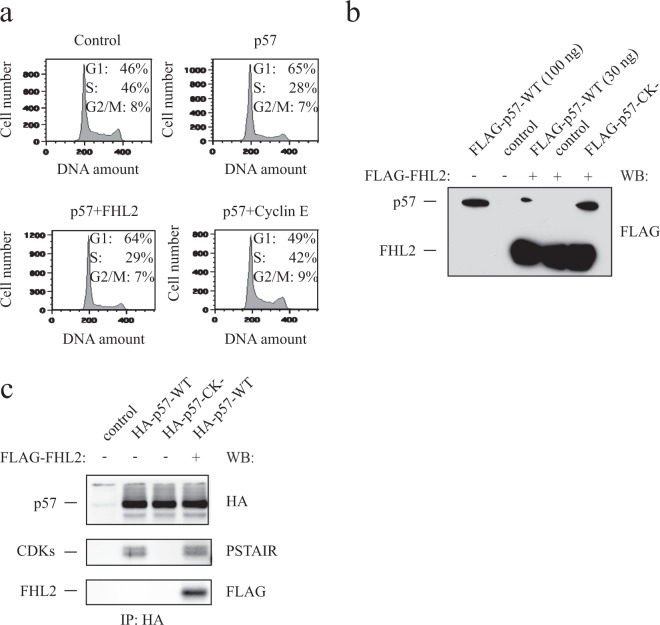
FHL2 does not regulate p57 cell cycle function. **(a)** The p57-induced G1-arrest is not reversed by FHL2. 293 T were transfected with p57 and/or FHL2 coding plasmids and cell cycle distribution of transfected cells was analysed by flow cytometry of propidium iodine stained cells. Distribution of cells in the different cell cycle phases (G1, S, G2/M) are indicated. **(b)** FHL2 expression exceeds p57 expression. Western blot analysis of extracts derived from 293 T cells transfected under the conditions for cell cycle analysis (30 ng pCruz-OctA-FLAG-p57 and 200 ng pCruz-OctA-FHL2) **(a)**. 30 or 100 ng of pCruz-OctA-FLAG-p57 were transfected in combination with 200 ng pCruz-OctA-FHL2 and levels of FLAG-fusion proteins detected by Western blotting with FLAG-antibodies. **(c)** FHL2 does not interfere with binding of p57 to CDKs. 293 T cells were transfected with plasmids expressing HA-tagged p57 wildtype (HA-p57-WT), HA-p57-CK-binding mutant (p57-CK-) or HA-p57-WT together with a FLAG-tagged FHL2 expressing vector. Extracts were subjected to HA-IP and bound FHL2 or CDKs detected by Western blotting using anti-FLAG and anti-PSTAIR antibodies. Signals corresponding to p57, FHL2 and endogenous PSTAIR-reactive CDKs are indicated left to the blot. Note that the cyclin/CDK binding deficient mutant (p57-CK−) does not bind to CDKs.

Binding of FHL2 to the N-terminal CDK-inhibitory domain might interfere with cyclin/CDK binding of p57 and therefore impair the function of p57 as a cell cycle inhibitor. To test this hypothesis, we transfected 293 T cells with p57. As expected, expression of p57 led to an increase of cells in G1 phase and to a decrease of cells in S-phase. However, co-transfection of p57 and FHL2 did not alter the p57-induced accumulation of cells in G1 phase (Fig. 4a), even when the amount of FHL2 protein strongly exceeded the levels of p57 (Fig. 4b). As a positive control, co-expression of cyclin E could revert the p57-induced cell cycle arrest (Fig. 4a). Furthermore, we analysed whether binding of p57 to cyclin/CDK complexes might become impaired by FHL2. We expressed HA-p57 in presence or absence of FHL2 in 293 T cells, immunoprecipitated p57 and analysed its binding to FHL2 and endogenous CDKs. We observed that p57 bound similar amounts of PSTAIR-reactive CDKs in the absence or presence of co-expressed FHL2 (Fig. 4c), indicating that FHL2 does not revert the p57-induced cell cycle arrest as a consequence of not interfering with p57 binding to cyclin/CDK complexes. Binding of FHL2 to the N-terminus of p57 may either not compete with p57 binding to cyclin/CDK complexes or be restricted to non CDK-bound p57.

### p57 activates FHL2 dependent promoters

**Figure 5:**
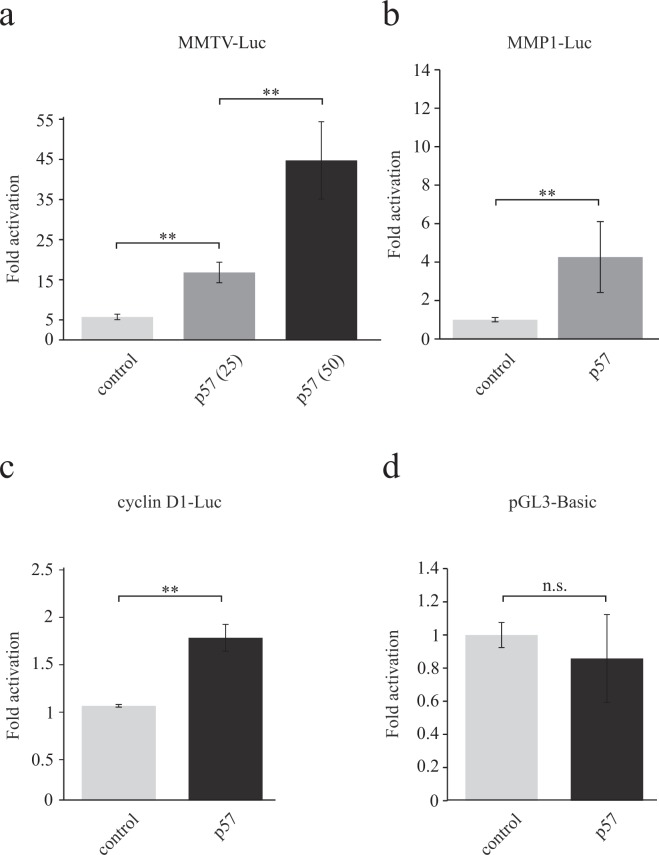
p57 activates FHL2-dependent reporter genes. **(a)** p57 increases the androgen-response of a mouse mammary tumor virus (MMTV) reporter gene in a dose-dependent manner. 293 cells were transfected with a MMTV-luciferase reporter, human androgen receptor and increasing amounts of p57 expressing plasmid. Four hours after transfection cells were treated for 24 hours with 10^−9^ M of the synthetic androgen R1881 or 70% Ethanol as vehicle control before harvesting. Extracts were processed and luciferase activities determined as described in Materials and Methods. Renilla normalised luciferase activities of R1881 treated cells are expressed as fold activation relative to control-treated cells. **(b)** p57 activates the human collagenase MMP-1 promoter. HeLa cells were transfected with a luciferase reporter gene under the control of the human collagenase promoter (MMP1-Luc) with (p57) or without p57-expressing plasmid. 40 hours after transfection cell extracts were prepared and processed as described above. Renilla normalised luciferase activities from the control transfection were set to one and others expressed as “fold activation” by p57. **(c)** p57 induces the activity of the human cyclin D1-promoter. HeLa cells were transfected with a luciferase reporter gene controlled by the human cyclin D1-promoter (cyclin D1-Luc) with (p57) or without a plasmid expressing p57. Cell extracts were prepared 40 hours after transfection and luciferase activities determined and expressed as “fold activation” as described before. **(d)** p57 does not affect a “backbone” reporter construct. The control pGL3-basic reporter construct was transfected into HeLa cells and activity determined upon p57 expression as above. Data are presented as the mean of three **(c)**, four **(a)** or five **(b**,**d)** independent experiments including standard deviation as error bars. (*)P < 0.05, (**)P < 0.01, (n.s.): not significant.

We next investigated whether FHL2 activity might be regulated by p57. FHL2 has been described as a coactivator for several transcription factors like the androgen receptor, AP-1 or β-catenin^21,34,35^. In these studies, reporter gene experiments demonstrated that FHL2 induces promoter activities by coactivating transcription factors. We examined a potential transcriptional regulation of the androgen-responsive MMTV-^36^, the AP-1 regulated human collagenase (MMP1)-^34^ and the β-catenin controlled cyclin D1-luciferase reporter genes^37,38^ by p57. We found that p57 substantially induced all three FHL2-regulated reporter genes (Fig. 5a–c). The activity of the hormone-induced MMTV reporter was increased by p57-expression by approximately 3- and 8-fold in a dose-dependent manner (Fig. 5a), the MMP1 reporter gene was approximately 4-fold induced by p57 (Fig. 5b) and the human cyclin D1 reporter was activated by 1.7-fold (Fig. 5c). The activity of the basal promoter construct pGL3-Basic was not markedly altered by p57 (Fig. 5d). These data indicate that p57 is able to activate promoters of several genes previously reported to be regulated by the coactivator FHL2. We therefore speculated that p57 might enhance the coactivator function of FHL2.

### p57 enhances the intrinsic transcriptional activity of FHL2

The reporter constructs tested suggest a role of p57 in the control of promoter activities.

**Figure 6:**
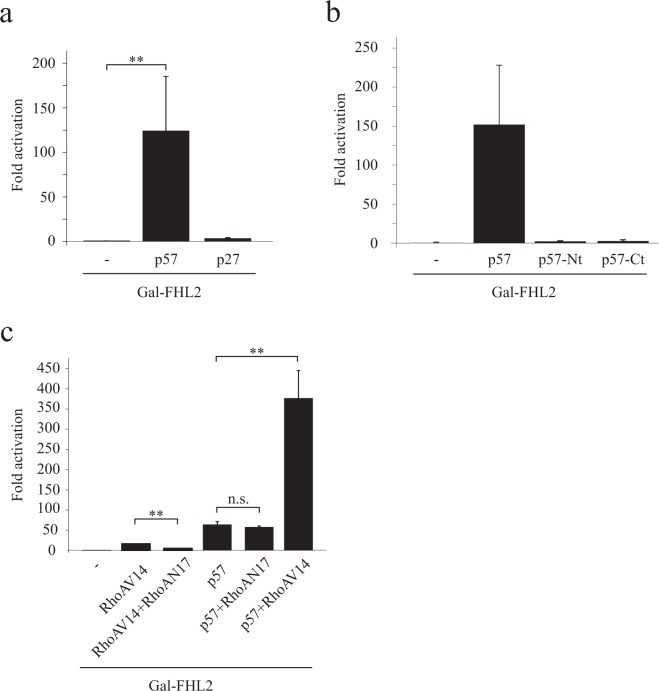
p57 increases the autonomous coactivator activity of FHL2 in a RhoA independent manner. **(a)** p57 strongly induces the autonomous transactivation function of FHL2. 293 cells with a genome-integrated Gal4-dependent firefly luciferase reporter gene (293FR) were transfected with a Gal-FHL2 expressing plasmid and plasmids expressing p57 or p27. As an internal control for transfection efficiencies a Renilla luciferase coding plasmid was included in all transfections. Renilla normalised firefly values of Gal-FHL2 transfected cells were set to one and values from p57 or p27 co-transfected cells are expressed relative to Gal-FHL2. **(b)** Full-length p57 is required for FHL2 activation. 293FR cells were transfected with Gal-FHL2 alone or in combination with plasmids coding for p57 or the indicated mutants and further processed like in (**a)**. Renilla normalised values for Gal-FHL2 were set to one and values from p57, p57-Nt or p57-Ct expressing cells are shown relative to Gal-FHL2. **(c)** p57 activation of Gal-FHL2 is independent of RhoA signaling. 293FR cells were transfected with Gal-FHL2 and the indicated plasmids coding for p57 and two mutants of RhoA, one constitutive active (“RhoAV14”) and the second dominant negative to RhoA signaling (“RhoAN17”). Transfected cells were further processed like in **a** and **b**. Gal-FHL2 values were set to one and the results expressed relative to it as “Fold activation”. Data are shown as the mean of three independent experiment, standard deviation is included as error bars. (*)P < 0.05, (*)*P < 0.01, (n.s.) non-specific.

FHL2 contains an intrinsic (also termed autonomous) transactivation activity^21,39^. At present still uncharacterised sequences in the FHL2 protein might be able to initiate transcription by interacting with the basal transcriptional machinery. While a direct biochemical confirmation of such a mechanism is missing, it is supported by experiments using artificial reporter gene systems^21^. To exclude the contribution of potential additional transcription factors, we employed such a reporter gene system, where the activity of the reporter solely depends on FHL2. This reporter is based on a Gal4-FHL2 fusion protein which consists of the DNA binding domain of the yeast transcription factor Gal4 fused in frame to the full-length human FHL2. 293 cells stably expressing the Gal4-DNA-binding domain (DBD) - dependent firefly luciferase reporter construct pFR-Luc (293FR) were transfected with plasmids encoding the fusion protein Gal-FHL2 alone or in combination with p57 or p27. Strikingly, we observed a strong activation of Gal-FHL2 activity by p57 (Fig. 6a), indicating a direct contribution of p57 to FHL2 coactivator activity. This strong activation is specific for p57, since the closely related p27 only marginally augmented FHL2 activity (Fig. 6a). Since the N-terminal domain of p57 binds FHL2 (Fig. 2d), we tested whether this domain might be sufficient to function as an FHL2 activator.

Interestingly, neither the N-terminal nor the C-terminal p57 domain could activate Gal-FHL2 (Fig. 6b). This suggests that binding to FHL2 is necessary but might not be sufficient for p57 to function as an FHL2 activator. Importantly, the expression of the N-terminal p57 domain was consistently lower compared to that of the C-terminal domain or the full-length protein (Fig. 2d and data not shown) and we were uncertain about matching expression levels. In addition, when the N-terminus of p57 is expressed as a YFP-fusion protein, a great portion of the protein localises to the cytoplasm (Supplementary Fig. S9). However, when the nuclear localization of the N-terminal p57 domain is enforced by attaching a nuclear localization sequence (NLS) of the SV40 large T-antigen (NLS1), we did not see any difference in Gal-FHL2 activation compared to the p57 N-terminal domain without NLS (Supplementary Fig. S9). Therefore, we conclude that full-length p57 is necessary to act as an activator for FHL2 in our reporter assays. The underlying mechanism of activation could be more complex and might require an extended N-terminal domain of p57.

### Induction of FHL2 coactivation function is independent of RhoA

Nuclear accumulation of FHL2 and also Gal-FHL2 activity can be induced by RhoA signaling. Transfection of cells with a constitutively active allele of RhoA (RhoA-V14) relocalised Gal-FHL2 from cytoplasmic focal adhesion sites to the nucleus, leading to the activation of a Gal4-dependent reporter gene^39^. We investigated whether Gal-FHL2 activation might be the consequence of p57-induced RhoA signaling. We transfected 293FR cells with Gal-FHL2 and p57 in presence or absence of a plasmid coding for a dominant negative allele of RhoA (RhoA-N17) and determined Gal-FHL2 activity in luciferase reporter assays. Whereas the activation of Gal-FHL2 by a constitutive active mutant of RhoA (RhoA-V14) was strongly reduced by co-expressing RhoA-N17, we found no considerable effect on p57 activation of Gal-FHL2 by blocking RhoA signaling (Fig. 6c). Interestingly, when RhoA-V14 and p57 were co-expressed, Gal-FHL2 was synergistically activated, supporting the hypothesis that p57 and RhoA might impinge on independent pathways of Gal-FHL2 activation.

### FHL2 activity might be repressed by high-molecular-weight complexes containing HDACs

**Figure 7:**
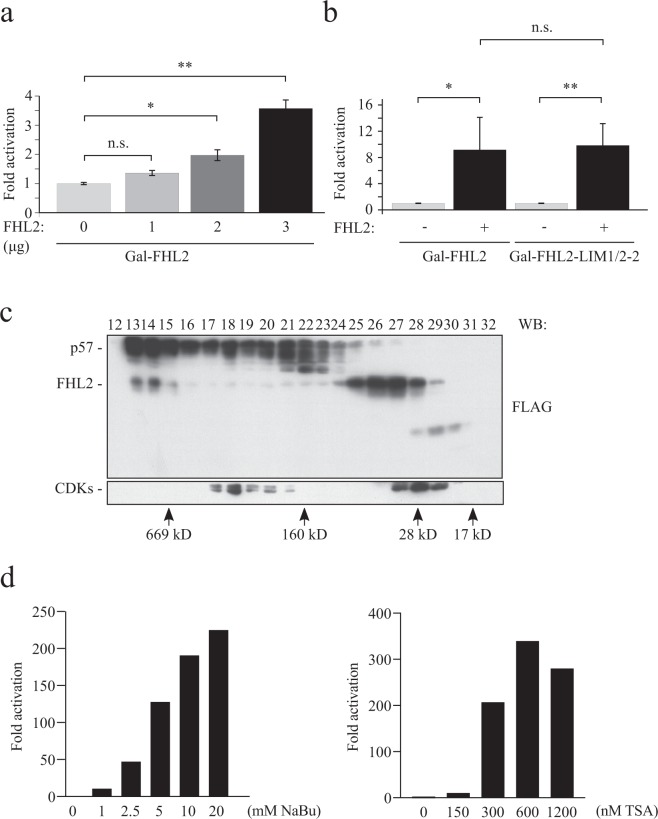
FHL2 is found in high molecular weight complexes and its activity is increased by HDAC inhibitors. **(a)** Evidence for a titratable repressor of FHL2 transcriptional activity. 293FR cells were transfected with Gal-FHL2 together with increasing amounts of a plasmid coding for FLAG-FHL2. A plasmid expressing renilla luciferase served as transfection control. Amounts of co-transfected FHL2-plasmid are indicated below the panel. Values obtained for control transfections (no FHL2, “0”) were set to one. Relative “fold activation” for FHL2 transfections is shown. Mean values, standard deviation and level of significance were determined from four independent experiments. **(b)** Dimerization-deficient FHL2 mutant is activated by FHL2. 293FR cells were transfected with Gal-FHL2 or the dimerization-defective mutant Gal-FHL2-LIM1/2–2 and a plasmid expressing FLAG-FHL2. The experiment was processed, analysed and the data presented as described in **a**. Mean values, standard deviation and level of significance were determined from four independent experiments. **(c)** p57 and FHL2 are components of high molecular weight complexes. Lysates from FLAG-tagged p57 and HA-tagged FHL2 transfected 293 T cells were applied to size exclusion chromatography. Fractionation by Superose 6 was followed by SDS-PAGE and Western blotting of the fractions indicated, using anti-FLAG and anti-HA antibodies. The elution profile of molecular weight markers is indicated below. Signals corresponding to FLAG-p57 and HA-FHL2 are indicated left from the blots. CDK proteins were detected using anti-PSTAIR antibodies. **(d)** HDAC-inhibitors sodium butyrate (NaBu) and Trichostatin A (TSA) induce Gal-FHL2 activity. 293FR cells were treated with indicated increasing amounts of NaBu or TSA for 24 h. Luciferase activities were determined as described above. Gal-FHL2 values of control-treated (water for NaBu and DMSO for TSA) were set to one and others expressed relative to it as “Fold activation”.

The potential need for full-length p57 in FHL2 regulation let us speculate that additional factors might be involved in this robust activation of Gal-FHL2. In addition to recruiting a potential activator of FHL2 coactivation, p57 might bind and inactivate an inhibitor of FHL2 coactivator function. FHL2 was reported previously to stimulate the transcriptional coactivator CBP/p300^37^. However, the association of FHL2 with corepressors has not been reported previously. Promoters can be silenced by chromatin remodeling factors, which often act in concert with additional proteins as multimeric complexes. c-Jun for instance was reported to be transcriptionally inactive although bound to its sites at promoters of regulated genes^40–42^. Initial evidence for the existence of such repressor molecules came from experiments where the effect of c-Jun expression in the Gal-c-Jun system was tested. It was shown that wildtype c-Jun, which cannot bind to the Gal4-sites in the Gal-reporter gene, increased Gal-c-Jun transactivation. From these experiments it was concluded that putative repressors are sequestered by c-Jun leading to a derepression of Gal-c-Jun^40^. We speculated that also Gal-FHL2 activity might be regulated by an unknown repressor. In a similar approach we investigated the effect of wildtype FHL2 expression on Gal-FHL2 transactivation. To rule out increased transactivation by dimerization of Gal-FHL2 with co-expressed FHL2, we included an FHL2 mutant consisting of the first half and two full LIM-domains (Gal-FHL2-LIM1/2-2). This mutant does not to bind to the full-length FHL2 protein^43,44^. Interestingly, Gal-FHL2 activity was induced by increasing amounts of co-expressed wildtype FHL2 (Fig. 7a) suggesting the association of FHL2 with factors repressing FHL2 coactivator activity. Notably, the dimerization-deficient Gal-FHL2-LIM1/2-2 mutant was activated by wildtype FHL2 to a similar degree as Gal-FHL2 (Fig. 7b) excluding dimerization as a mechanism for increased transactivation.

We next investigated if p57 and FHL2 might be components of larger molecular weight repressor complexes and determined the distribution of both proteins by size exclusion chromatography. Extracts from 293 T cells transfected with FLAG-tagged FHL2 and p57 were separated on a Superose 6 HR 10/30 column. Interestingly, substantial amounts of both p57 and FHL2 eluted in fractions with high molecular weight (>500 kD; Fig. 7c, fractions 13 to 15). These results are consistent with the role of FHL2 as a scaffolding protein^18^ and suggest that p57 and FHL2 are components of high-molecular-weight complexes and might interact with transcriptional repressors.

We speculated that one of such transcriptional repressors might be HDACs. HDACs repress transcription typically by deacetylating histones at the site of promoters. Such promoters are activated by small molecules which bind to HDACs and inhibit their catalytic activity (HDAC inhibitors, HDACis). To elucidate whether FHL2 coactivator activity is repressed by HDACs, we treated 293FR-cells with increasing amounts of the two HDACis sodium butyrate (NaBu) or Trichostatin A (TSA) and determined FHL2 coactivator activities. We found that both drugs induced Gal-FHL2 activity in a dose-dependent manner (Fig. 7d), indicating that Gal-FHL2 in untreated cells is repressed by HDACs or by a repressor complex containing HDAC enzymes.

### FHL2 is in a complex with HDACs and p57 interferes with the binding of HDACs to FHL2

**Figure 8:**
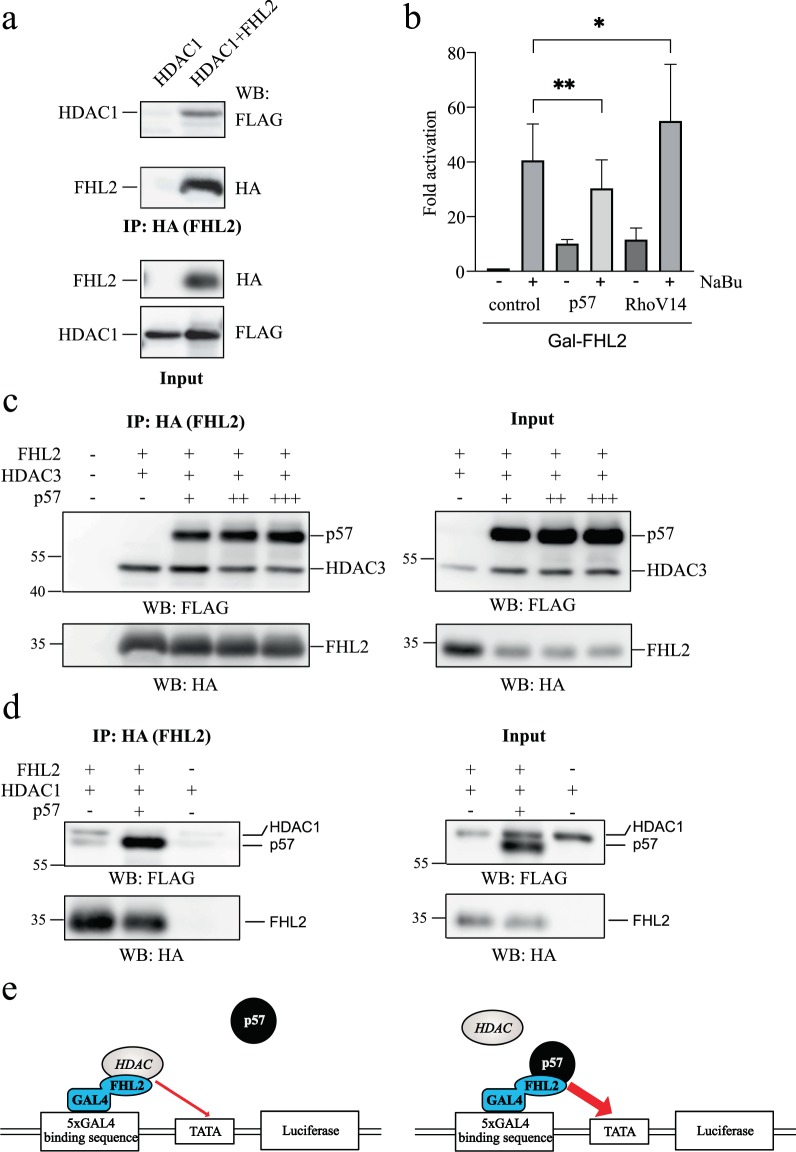
HDAC1/3 and p57 bind FHL2 in a competitive manner. **(a)** FHL2 binds histone deacetylase 1 (HDAC1). Western blot results after HA-IP of FHL2. 293 cells were cotransfected with plasmids expressing FLAG-tagged HDAC1 and HA-FHL2. FHL2 was immunoprecipitated with anti-HA antibodies and coprecipitated FLAG-HDAC1 was detected by Western blot using anti-FLAG-specific antibodies. “Input” controls for FLAG-HDAC1 expression in extracts used for IP. **(b)** Indication that p57 shares a common pathway with sodium butyrate (NaBu) in activating Gal-FHL2. 293 cells co-transfected with pFR-LUC and the indicated plasmids were treated with NaBu or left untreated. Firefly luciferase values were normalised to renilla luciferase obtained from a co-transfected Ubi-renilla plasmid. Normalised Gal-FHL2 values of control-transfected cells in the absence of NaBu were set to one and others expressed relative to it as “Fold activation”. **(c,d)** p57 competes with HDAC3 and HDAC1 for FHL2 binding. Western blot analysis of HA-IPs from transfected 293 T cells expressing HA-FHL2 and FLAG-HDAC3 plus increasing amounts of FLAG-p57 indicated: “+” = 1/1, “++” = 2/1 and “+++” = 3/1 ratio of p57 to HDAC3 expressing plasmids. **(c)** or co-transfected with a threefold excess of p57-expression plasmid compared to HDAC1 **(d)**. **(c)** Western blots were probed for p57 and HDAC3 with mouse FLAG antibody (upper panels) and for FHL2 with mouse HA antibody (lower panels); **(d)** Western blots were probed for p57 and HDAC1 with mouse FLAG antibody (upper panels) and for FHL2 with mouse HA antibody (lower panels). **(e)** Mechanistic model for p57 in activating Gal-FHL2 coactivator function. For details see text.

Since FHL2 activity is regulated by HDACis, we speculated that FHL2 might be in a complex with HDACs. To test this we expressed HDAC1, HDAC3 and FHL2 in 293 cells and analysed binding in immunoprecipitation experiments. Interestingly, HDAC1 and HDAC3 bound to FHL2 (Fig. 8a,c), indicating that FHL2 and HDACs form a complex *in vivo*. We wondered if FHL2 activation by p57 might be the consequence of interference with HDAC function. We analysed 293 cells, transfected with the Gal-dependent reporter FR-LUC and determined Gal-FHL2 activation by p57 in the presence or absence of the HDACi NaBu. Strikingly, we found rather an inhibition than a further activation of Gal-FHL2 by p57 in the presence of NaBu (Fig. 8b). This result is consistent with a common pathway of p57 with NaBu in activation of Gal-FHL2. Interestingly, activation of the Rho-pathway by RhoV14 expression further increased the activation by NaBu. This is in accordance with our observation that p57 and RhoA activate Gal-FHL2 by distinct pathways (Fig. 6c). If p57 inhibits HADC function on FHL2, p57 might compete with FHL2 binding. We tested this hypothesis in transient transfection experiments. 293 cells were co-transfected with HDAC3, FHL2 and increasing amounts of p57. Similar to HDAC1, HDAC3 also co-immunoprecipitated with FHL2. Consistent with our hypothesis, binding of HDAC3 to FHL2 was reduced by increased expression of p57 (Fig. 8c). In a second experiment, HDAC1, FHL2 and a threefold excess of p57 were expressed in 293 T cells. Strong expression of p57 markedly reduced binding of HDAC1 to FHL2 (Fig. 8d). Based on these observations one can propose a mechanistic model how p57 activates the intrinsic transactivation function of FHL2 (Fig. 8e). In this model FHL2 transcription function is reduced by HDACs or complexes containing HDAC activity. p57 competes with HDACs for FHL2 binding, and thereby HDAC mediated repression is relieved and FHL2 can stimulate promoter activity.

## Discussion

The role of p57 as a cyclin/CDK interacting protein is well established. Studies of transgenic mice revealed a prominent role of the cyclin/CDK-inhibitory function of p57^Kip2^ in reverting the severe developmental defects of p57-knock-out mice^8,9^. However, not all phenotypes were reverted, indicating that p57 might have important functions in addition to cyclin/CDK-inhibition.

Therefore, we applied a yeast two-hybrid screen and identified the transcription regulator four-and-a-half LIM-only protein 2 (FHL2) as a novel p57 interacting protein. Endogenous p57/FHL2 complexes were detected in the cervix carcinoma cell line HeLa and the colon carcinoma cell line HRT-18 which may indicate a general functional role of this interaction, not restricted to specific tissues or tumor cell types.

FHL2 was initially identified as a binding partner and transcription coactivator of the human androgen receptor^21^. However, in following studies FHL2 turned out as a multifunctional protein with binding partners involved in numerous cellular processes^18,20^. Despite of its role as a transcription regulator, FHL2 most likely does not directly bind to chromatin. As a coactivator or corepressor of transcription factors, FHL2 contains an intrinsic transactivation or transrepression activity^21,43,45^. Transcription factors like AP-1, β-catenin or the androgen receptor^21,34,35,43^ have been shown to be coactivated by FHL2. The molecular mechanisms of activation, however, is not understood in detail.

p57 activated expression of AP-1, β-catenin and androgen receptor dependent reporter genes, all established promoters of FHL2-regulated genes and strongly enhanced the intrinsic (autonomous) transactivation activity of Gal4-FHL2. Although the latter might not fully reflect p57 action at natural promoter sites *in vivo*, these results describe a so far unprecedented regulatory property of p57 in transcription.

When we investigated the possible mechanism of FHL2-activation by p57, we could rule out an indirect activation of FHL2 by Rho signaling. Activation of the RhoA pathway was shown previously under similar experimental conditions to relocalise FHL2 from the cytoplasm to the nucleus and to induce Gal-FHL2 activity up to 10-fold^39^. Interestingly, in previous reports another LIM-domain containing protein, the LIM-kinase-1 (LIMK-1), a downstream effector of Rho-signaling, was shown to bind p57^46,47^. Although the LIM-domain amino acid sequences between FHL2 and LIMK-1 are not similar {Kadrmas *et al*., 2004}, the secondary structure of both proteins might enable binding to p57. Interestingly, LIMK-1 binding to mouse p57 requires amino acids 93 to 260 (104–228 in human), a central region of p57 devoid of cyclin/CDK-binding and inhibition. We found a minimal FHL2-binding sequence in human p57 from amino acid 1 to 125 (1–115 in mouse) which contains the cyclin/CDK-binding domain. It would be interesting to examine whether the overlapping sequence of human p57 from 104 to 125 (or 93–114 in mouse) contains a docking site for LIM-domain containing proteins.

Based on several observations, an involvement of RhoA signaling in p57 dependent FHL2 activation is unlikely. First, we did not see changes in localization of FHL2 upon p57 co-expression in fluorescence microscopy, second, LIMK-1 expression did not activate Gal-FHL2^39^ and third and most importantly, blocking the RhoA pathway by a dominant negative RhoA-mutant did not reduce the activation of Gal-FHL2 by p57. Also, a constitutively active RhoA-mutant together with p57 synergistically activated Gal-FHL2, which is an expected result in case of two independent activation pathways affecting the same effector molecule^48^.

Transcription activators can be inactive even when bound to their promoter or enhancer sites. For example, the AP-1 transcription factor c-Jun is repressed by multiprotein-complexes containing histone deacetylases (HDACs)^40,42^. Binding and repression of c-Jun by HDAC3 was shown to be relieved upon titration of HDAC3 and c-Jun kinase (JNK)-dependent phosphorylation of c-Jun^40^. Several lines of experimental evidence support the idea that FHL2, at least at some promoters, might be repressed by a similar mechanism. First, Gal-FHL2 was activated by increasing amounts of wildtype FHL2, indicating the presence of a titratable repressor like HDAC3 for c-Jun. Second, we could detect binding of FHL2 to histone deacetylase 1 and 3 and, most strikingly, treatment of cells with the two histone deacetylase inhibitors sodium butyrate (NaBu) and Trichostatin A (TSA) strongly induced FHL2 activity.

The presence of multiple LIM-domains in FHL2 hints towards a scaffolding function of FHL2 in multimeric complexes^18^. Interestingly, it has already been reported that FHL2 interacts with and regulates components of chromatin remodeling complexes. By building a trimeric complex with the transcription factor β-catenin and the GNAT-family histone acetylase CBP/p300, FHL2 stimulates histone acetylation of β-catenin leading to activated transcription of Wnt-dependent genes like cyclin D1^35,37^. In addition, FHL2 was also shown to function as a corepressor of the transcription factor FOXO1^49^ by stimulating FOXO1 deacetylation by Sirtuin1 (SIRT1), a class III histone deacetylase. We show for the first time, that FHL2 binds to the transcriptional repressors HDAC1 and 3, however, given the high similarity of class I HDACs, more studies have to be conducted to determine the whole repertoire of histone deacetylases which may bind FHL2.

The molecular weight of chromatin remodeling complexes ranges from several hundred kD to more than two MDa. Our size exclusion chromatography experiments revealed the presence of ectopically expressed FHL2 in size fractions higher than 600 kD, indicating that FHL2 might be a component of such high-molecular-weight complexes. It will be an exciting challenge to determine endogenous multiprotein complexes which contain FHL2 and HDACs and to explore their functional role *in vivo*.

Our data are consistent with one potential mechanistic model, where Gal-FHL2 activity is silenced by histone deacetylases. The strong activation of FHL2-coactivator function by p57 might involve its interference with HDAC-activity. We also observed that p57 could not further stimulate activation of Gal-FHL2 in the presence of the histone deacetylase inhibitor sodium butyrate. Rather, activation by sodium butyrate was reduced in the presence of p57. This result is consistent with the model that p57 is bound to FHL2 and thereby hinders HDACs from binding and repressing FHL2-dependent transcription. We could demonstrate reduced binding of HDAC1 and 3 to FHL2 in the presence of increasing amounts of p57 in co-immunoprecipitation experiments, indicating a competition of p57 and HDACs for binding to FHL2. However, the induction of Gal-FHL2 activity by p57 is much stronger compared to that obtained by titration with FHL2. This substantial difference might be the result of p57 binding to Gal-FHL2. p57 binding to FHL2 might shield from HDAC rebinding or might attract positive transcriptional regulators like histone acetylases. Based on the FHL2/p57-binding experiments, one could speculate that the N-terminus of p57 binds to FHL2 and the C-terminus of p57 might serve as a docking site for transcriptional activators. Such a model is supported by the potential need for full-length p57 to activate FHL2 and its association with high molecular weight complexes. Interestingly, recombinant p57 was also recently found to bind to the class II histone deacetylase HDAC7 in a protein array^8^. Binding of p57 to HDACs might also explain the repression of neurogenic transcription factors like Mash1, NeuroD and Nex/Math2 in neuronal differentiation (Joseph *et al*., 2009). Interestingly, the same authors demonstrated transcriptional repression by a Gal4-DBD-p57 fusion protein in a Gal4-dependent reporter system. It is tempting to speculate that also p57 might be a regulatory component of multiprotein complexes composed of FHL2, HDACs and other chromatin remodeling factors. It will be exciting to determine the components of large p57 and FHL2 containing protein complexes and to reveal their physiological roles, including pathophysiological processes like tumor initiation and promotion.

A role for p57 in transcription control has been uncovered earlier. p57 can inhibit the phosphorylation of the myogenic transcription factor MyoD, which leads to a stabilization of MyoD and activation of gene-promoters involved in myogenesis^15^. Intriguingly, also for FHL2 a role in promoting myogenesis has been reported^43^. Ectopic expression of FHL2 induced differentiation of the mouse myoblast cell line C2C12. It is reasonable to speculate that increasing levels of p57 in C2C12 upon differentiation^15^ might induce the transcription activity of FHL2 and thereby contribute to differentiation. Whether this shared role of p57 and FHL2 in myogenesis depends on the interaction of both proteins and if p57 acts as a positive regulator of FHL2 transcription activity in the regulation of myogenic genes is an important question and needs to be investigated in the future.

The finding that p57 regulates the transcriptional activity of FHL2 and FHL2-regulated genes clearly represents a novel function of p57^Kip2^ in addition to cell cycle control. It will be challenging and exciting to examine the contribution of p57 to transcription programs. This might be also relevant for a better understanding of cancerogenesis and tumorigenesis. It is tempting to speculate that p57, due to its interaction with transcription factors like FHL2 or as a component of other larger transcription complexes might modulate the activity of a broader spectra of transcription factors.

## Supplementary information

Supplementary Information.
