# Supplementary material for: The CDK inhibitor p57Kip2 enhances the activity of the transcriptional coactivator FHL2
Source: Sci Rep. 2020 Apr 28;10:7140. doi: 10.1038/s41598-020-62641-4 (PMC7188849; doi:10.1038/s41598-020-62641-4)
Supplement: Supplementary file 1 — Supplementary Information. [file 41598_2020_62641_MOESM1_ESM.pdf]

The CDK inhibitor p57^{Kip2} enhances the activity of the transcriptional coactivator FHL2

Michael Keith Kullmann, Silvio Roland Podmirseg, Martina Roilo and Ludger Hengst

Supplementary information: supplementary material, methods and figures:

Supplementary material

Plasmids and oligonucleotide sequences. The template for initial p57 clonings was a p57 cDNA in the Bluescript vector pBS-KSII (+)[1]. The yeast expression vector pAS2-1-p57 and the bacterial expression vector pET28a-p57 were obtained by amplifying p57 from the pBS-KSII plasmid with the primer pair p57-NdeI-for: AGCCCCGGCATATGTCCGA / M13-rev: CAGGAAACAGCTATGACCATG. The amplicon was digested with NdeI/EcoRI and inserted into NdeI/EcoRI digested pAS2-1 (Clontech) and pET28a+ (Novagen). pAS2-1-p21 and pAS2-1-p27 were obtained by inserting the NdeI/EcoRI-fragments from pET28a-p21 and pET28a-p27[2] into pAS2-1. For initial clonings of FHL2 constructs an FHL2 expressing plasmid isolated from the yeast two-hybrid screen (pACT2-FHL2) was used. pBS-FHL2 was obtained by insertion of the FHL2 ORF into pBS-SK (Stratagene, La Jolla, CA, USA). Cyclin D3 was expressed from plasmid pAR7[3]. Mammalian FHL2 expressing vector pCMX-FHL2, the human androgen receptor pSG5hAR and the androgen-responsive reporter MMTV-Luc were already described before[4]. Mammalian expression vectors for hemagglutinin-tagged (HA) p21, p27 and p57 pCruz-HA-p21, pCruz-HA-p27, pCruz-HA-p57 and pCruz-HA-FHL2 were obtained by inserting p21, p27 or p57 ORF into the multiple cloning site of pCruz HA and FLAG-tagged pCruz-OctA-FHL2 and pCruz-OctA-p57 into the multiple cloning site of pCruz-OctA (Santa Cruz). Except results presented in Fig. 2b and 6c, all expression vectors for p57, p57 mutants, p27, p21, FHL2 and FHL2 mutants were generated by Gateway-cloning (Invitrogen). p27 and p21 gateway cloning was already described elsewhere[5], p57 and FHL2 ORFs were amplified by two step PCR with appropriate primer pairs. First PCR adapter primers were attB1: GGGGACAAGTTTGTACAAAAAAGCAGGCTCC, attB2: GGGGACCACTTTGTACAAGAAAGCTGGGTC. Template specific primer pairs were attB1-p57: ACAAAAAAGCAGGCTCCATGTCCGACGCGTCCCTCCGCAGC / attB2-p57: CAAGAAAGCTGGGTCTCACCGCAGCCTCTTGCGCGGGG for cloning pDONR207-p57 and attB1-fhl2: ACAAAAAAGCAGGCTCCATGACTGAGCGCTTTGACTG / attB2-fhl2: CAAGAAAGCTGGGTCTTAAATGTCTTTCCCACAGTC for cloning pDONR207-FHL2, attB1-p57: ACAAAAAAGCAGGCTCCATGTCCGACGCGTCCCTCCGCAGC / attB2-p57

(125): CAAGAAAGCTGGGTCTTAGGACTCAGCGGCCGGCTCG for cloning pDONR207-p57-Nt, attB1-p57 (126): ACAAAAAAGCAGGCTCCCTCGACGGCCTCGAGGAGGCGCCGG / attB2-p57: CAAGAAAGCTGGGTCTCACCGCAGCCTCTTGCGCGGGG for cloning pDONR207-p57-Ct and attB1p57: ACAAAAAAGCAGGCTCCATGTCCGACGCGTCCCTCCGCAGC / attB2-p57-NLS1 (125): CAAGAAAGCTGGGTCTTATCCTACCTTTCTCTT CTTTTTTGGGGACTCAGCGGCCGGCTCG for cloning pDONR207-p57-Nt-NLS1, attB1-fhl2: ACAAAAAAGCAGGCTCCATGACTGAGCGCTTTGACTG / attB2-fhl2 (95): CAAGAAAGCTGGGTCTTAGTTGGAATAGCAGTCTGTACAG for cloning pDONR207-FHL2-LIM1/2-1, attB1-fhl2: ACAAAAAAGCAGGCTCCATGACTGAGCGCTTTGACTG / attB2-fhl2 (157): CAAGAAAGCTGGGTCTTATTGTTTCTCATAGCAGGGCACAC for cloning pDONR207-FHL2-LIM1/2-2, attB1-fhl2 (96): ACAAAAAAGCAGGCTCCGAGTACTCATCCAAGTGCCAGG / attB2-fhl2: CAAGAAAGCTGGGTCTTAAATGTCTTTCCCACAGTC for cloning pDONR207-FHL2-LIM2-4, attB1-fhl2 (158): ACAAAAAAGCAGGCTCCCATGCCATGCAGTGCGTTCAG / attB2-fhl2: CAAGAAAGCTGGGTCTTAAATGTCTTTCCCACAGTC for cloning pDONR207-FHL2-LIM3-4, attB1-fhl2 (96): ACAAAAAAGCAGGCTCCGAGTACTCATCCAAGTGCCAGG / attB2-fhl2 (217): CAAGAAAGCTGGGTCTTAATACAAGTCACAGAAGCAG for cloning pDONR207-FHL2-LIM2-3. Second PCR products were recombined in the BP reaction into the donor vector pDONR207 (Invitrogen), the isolated plasmids verified by sequencing and used in the LR reaction to obtain fluorescent protein or epitope tagged p57 and FHL2 expressing constructs pEYFP-p57, pCherry-FHL2, pDEST-3xHA-p57, pDEST-3xHA-FHL2, pDEST-3xFLAG-p57, pDEST-3xFLAG-FHL2 and p57 amino- and carboxyterminal domain- and the FHL2 LIM-domain mutants expressing vectors pDEST-3xHA-p57-Nt and pDEST-3xHA-p57-Ct, pDEST-3xFLAG-FHL2-LIM1/2-1, pDEST-3xFLAG-FHL2-LIM2-4, pDEST-3xFLAG-FHL2-LIM3-4 and pDEST-3xFLAG-FHL2-LIM2-3. The -962 human cyclin D1 promoter pGL3-Basic plasmid ("cyclin D1-Luc") was a gift from Frank McCormick (Addgene plasmid # 32727) and described earlier[6], the -517/+63 human matrix metalloprotease 1 promoter pGL3-Basic plasmid ("MMP1-Luc") was kindly provided by Hans van Dam and originated from a corresponding CAT reporter gene[7]. pGL3-Basic is a control backbone reporter (Promega, Madison, WI, USA). The pFR-Luc reporter plasmid contains a synthetic promoter with five tandem repeats of the yeast GAL4 binding sites in front of a firefly luciferase gene (Stratagene, La Jolla, CA, USA). The ubiquitin promoter-

driven Renilla luciferase reporter construct pUbi-Rluc controls and normalises for transfection efficiency[8]. The Gal-FHL2 wildype and Gal-FHL2-LIM1/2-2 mutant expressing plasmids pSG424-FHL2 and pSG424-FHL2-LIM1/-2 were cloned by using the primer pairs hsfhl2+BamHI-for: TTTTGGATCCTGACTGAGCGCTTTGACTG / hsfhl2+SacI-rev: TTTTGAGCTCTTAAATGTCTTTCCCACAGTC and hsfhl2+BamHI-for: TTTTGGATCCTGACTGAGCGCTTTGACTG / M325+SacI-rev: TTTTGAGCTCTTATTGTTTCTCATAGCAGGGC which introduce a BamHI site at the 5´- and a SacI site at the 3´-end of the amplicons. PCR-products were BamHI/SacI digested and ligated into BamHI/SacI digested pSG424. RhoA active and dominant negative mutants expressing plasmids pEF-RhoAV14 and pEF-RhoAN17 have been described before[9]. The human histone deacetylase 1 and 3 expressing constructs HDAC1/3 FLAG were a gift from Eric Verdin (Addgene plasmid # 13820/13819) and has been described earlier[10]. The plasmid pBB14 used for cell cycle analysis of transfected cells was a gift from Lynn Enquist (Addgene plasmid # 18657) and is described elsewhere[11]. Rc/CMV cyclin E was a gift from Bob Weinberg (Addgene plasmid # 8963) and has been described earlier[12].

Supplementary methods

Subcellular fractionation of 293 cells. Cytosolic and nuclear fractions of 293 cells were generated by the REAP-method[13] with only minor modifications. Briefly, a semiconfluent culture of transfected 293 cells was collected from a 10 cm cell culture plate by scraping and washed twice with PBS. The cell pellet was resuspended in 600 µl of REAP-buffer consisting of 0.1% IGEPAL CA-630 (Sigma-Aldrich, St. Louis, MO, USA -Aldrich, St. Louis, MO, USA) in PBS. For whole cell lysate 300 µl of cell suspension was immediately added to 300 µl 2x Laemmli extraction buffer containing 100 mM DTT and homogenized using an ultrasonic homogeniser (Sonoplus, Bandelin, Berlin, Germany). Remaining cell suspension was centrifuged for approximately 10 sec. until reaching 10000 g ("pop-spin"). Supernatant, which corresponds to the cytoplasmic fraction, was carefully removed and added to 300 µl 2x Laemmli extraction buffer containing 100 mM DTT. Finally, after thoroughly removing residual liquid, the nuclear pellet was extracted and homogenized in 300 µl 2x Laemmli extraction buffer containing 100 mM DTT using an ultrasonic homogeniser (Sonoplus, Bandelin, Berlin, Germany). Equal volumes of the three fractions were resolved by SDS-PAGE followed by immunoblotting using respective antibodies.

1. Matsuoka, S. *et al.* p57KIP2, a structurally distinct member of the p21CIP1 Cdk inhibitor family, is a candidate tumor suppressor gene. *Genes Dev.* **9,** 650–662 (1995).
2. Grimmler, M. *et al.* Cdk-inhibitory activity and stability of p27Kip1 are directly regulated by oncogenic tyrosine kinases. *Cell* **128,** 269–280 (2007).
3. Motokura, T., Keyomarsi, K., Kronenberg, H. M. & Arnold, A. Cloning and characterization of human cyclin D3, a cDNA closely related in sequence to the PRAD1/cyclin D1 proto-oncogene. *J. Biol. Chem.* **267,** 20412–20415 (1992).
4. Müller, J. *et al.* FHL2, a novel tissue-specific coactivator of the androgen receptor. *Embo J* **19,** 359–369 (2000).
5. Podmirseg, S. R. *et al.* Caspases uncouple p27(Kip1) from cell cycle regulated degradation and abolish its ability to stimulate cell migration and invasion. *Oncogene* **35,** 4580–4590 (2016).
6. Tetsu, O. & McCormick, F. Beta-catenin regulates expression of cyclin D1 in colon carcinoma cells. *Nature* **398,** 422–426 (1999).
7. Angel, P. *et al.* 12-O-tetradecanoyl-phorbol-13-acetate induction of the human collagenase gene is mediated by an inducible enhancer element located in the 5'-flanking region. **7,** 2256–2266 (1987).
8. Roilo, M., Kullmann, M. K. & Hengst, L. Cold-inducible RNA-binding protein (CIRP) induces translation of the cell-cycle inhibitor p27Kip1. *Nucleic Acids Research* **46,** 3198–3210 (2018).
9. Kampfer, S. *et al.* Protein Kinase C Isoforms Involved in the Transcriptional Activation of Cyclin D1 by Transforming Ha-Ras. *J. Biol. Chem.* **276,** 42834–42842 (2001).
10. Emiliani, S., Fischle, W., Van Lint, C., Al-Abed, Y. & Verdin, E. Characterization of a human RPD3 ortholog, HDAC3. *Proceedings of the National Academy of Sciences* **95,** 2795–2800 (1998).
11. Brideau, A. D., Banfield, B. W. & Enquist, L. W. The Us9 gene product of pseudorabies virus, an alphaherpesvirus, is a phosphorylated, tail-anchored type II membrane protein. *Journal of Virology* **72,** 4560–4570 (1998).
12. Hinds, P. W. *et al.* Regulation of retinoblastoma protein functions by ectopic expression of human cyclins. *Cell* **70,** 993–1006 (1992).
13. Suzuki, K., Bose, P., Leong-Quong, R. Y., Fujita, D. J. & Riabowol, K. REAP: A two minute cell fractionation method. *BMC Research Notes* **3,** 294 (2010).

Supplementary figures

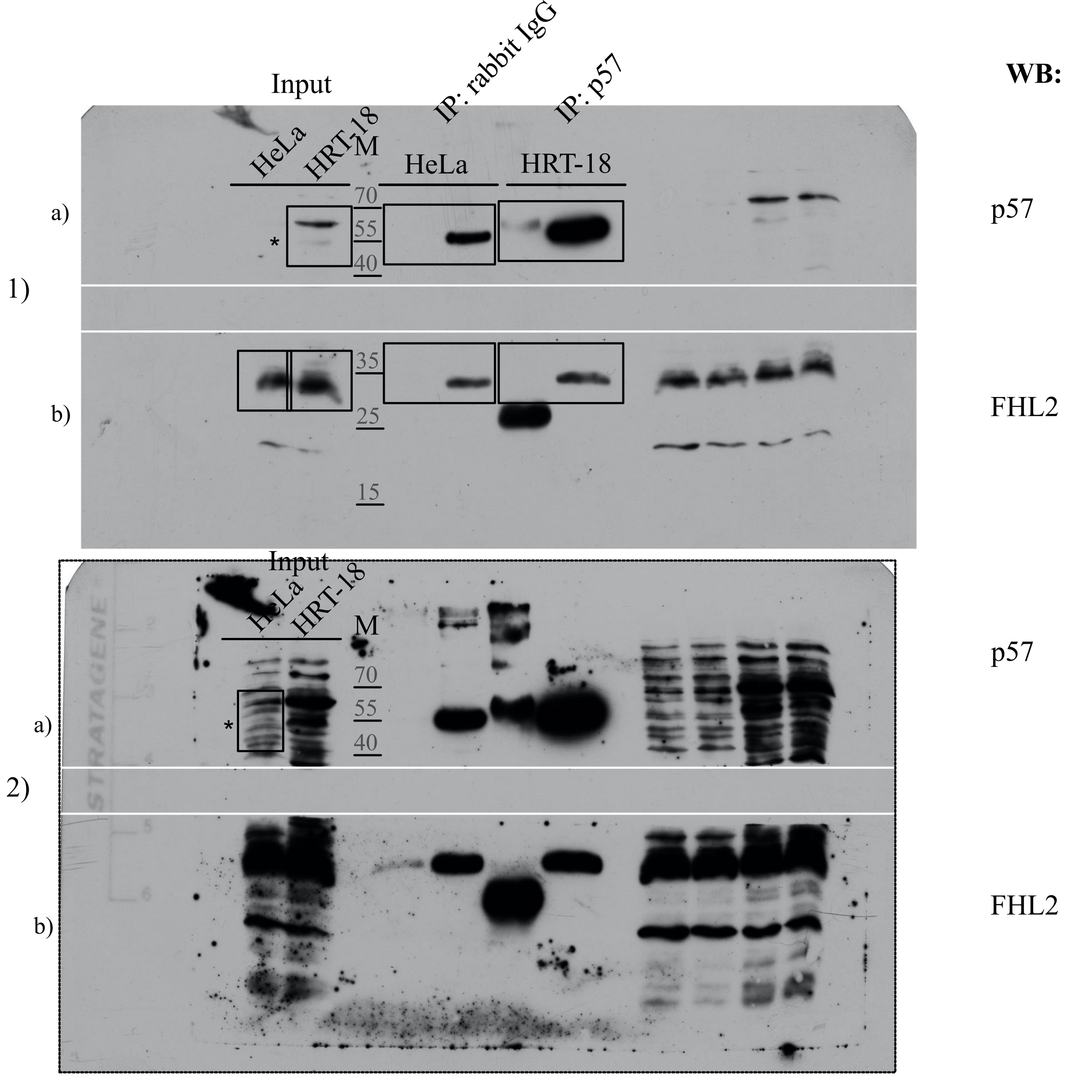

Supplementary Figure S1: original scans from immunoblots, approximate cropped areas are indicated by rectangles and were used for the figures in the main article. Lower scan (2) represents a long exposure from which the cropped area should visualize expression of p57 in HeLa cells. Asterisk (*) indicate band corresponding to p57. White horizontal lines indicate the lower (immunoblot for p57) and upper (immunoblot for FHL2) boundaries of two separate PVDF membranes (a and b) exposed to the same sensitive film. Processed for Fig. 1B in main article. Boundaries of the TIFF-scans are marked by black dashed rectangles.

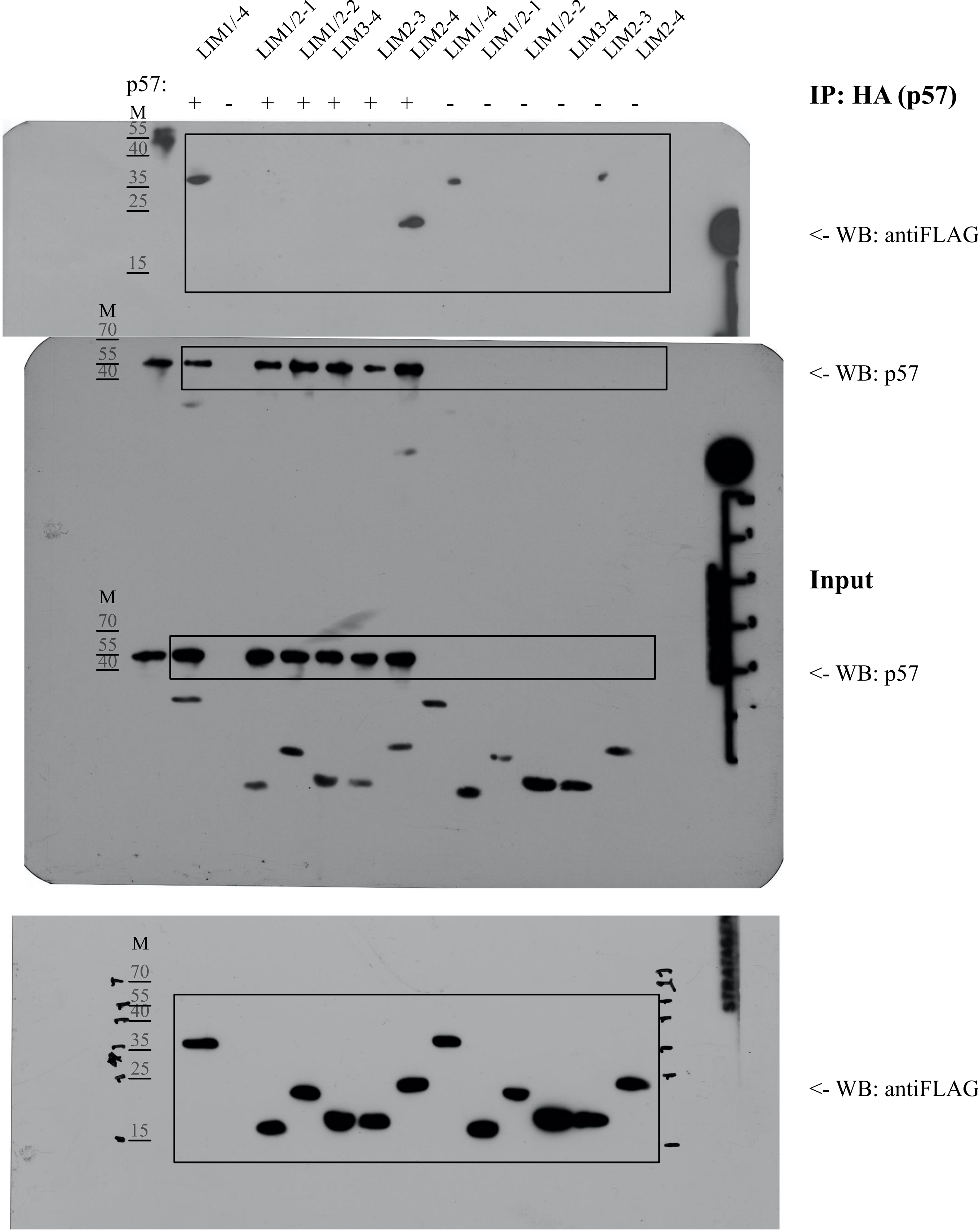

Supplementary Figure S2: original scans from immunoblots, approximate cropped areas are indicated by rectangles. Processed for Fig. 2b in article.

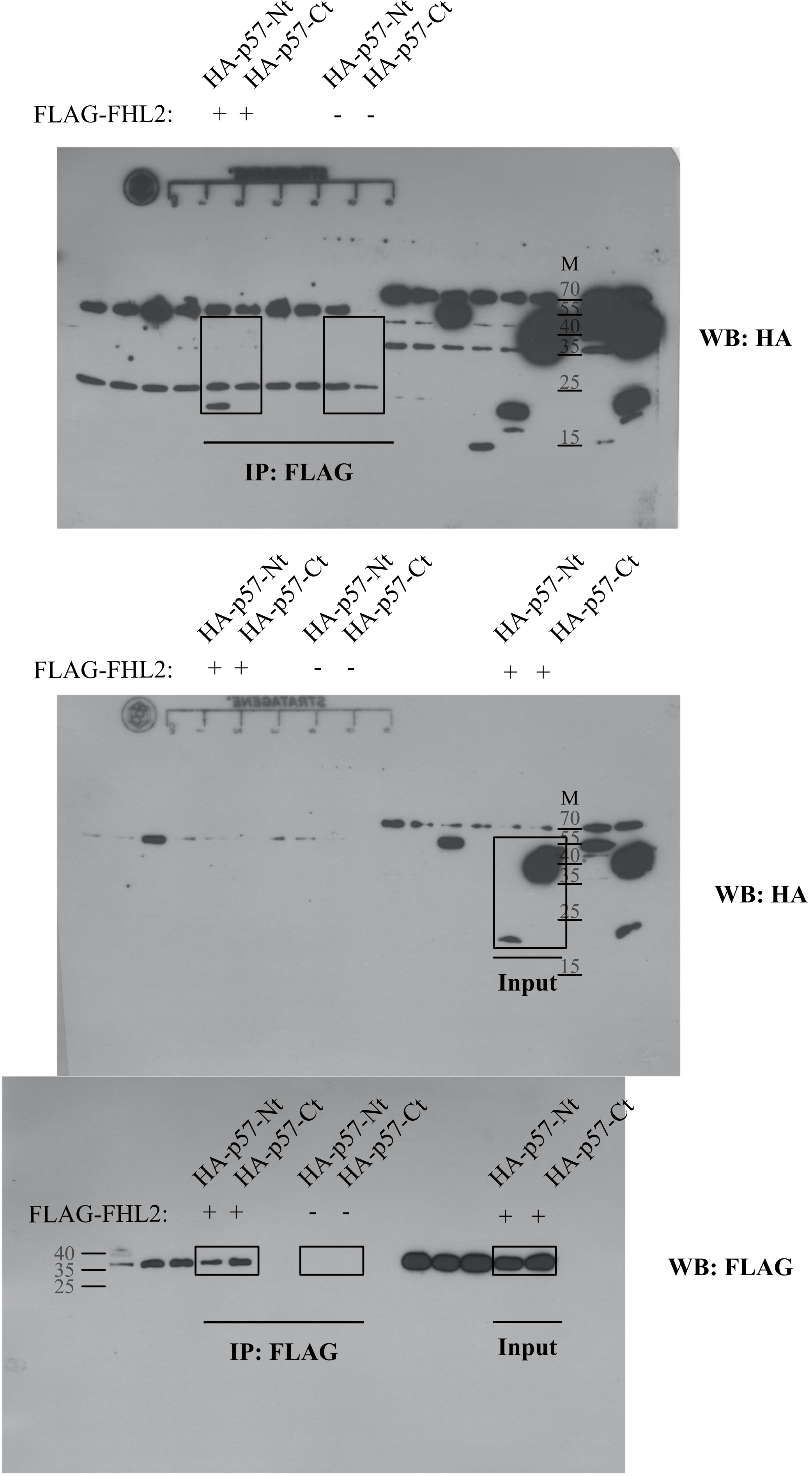

Supplementary Figure S3: original scans from immunoblots, approximate cropped areas are indicated by rectangles. Processed for Fig. 2d in article.

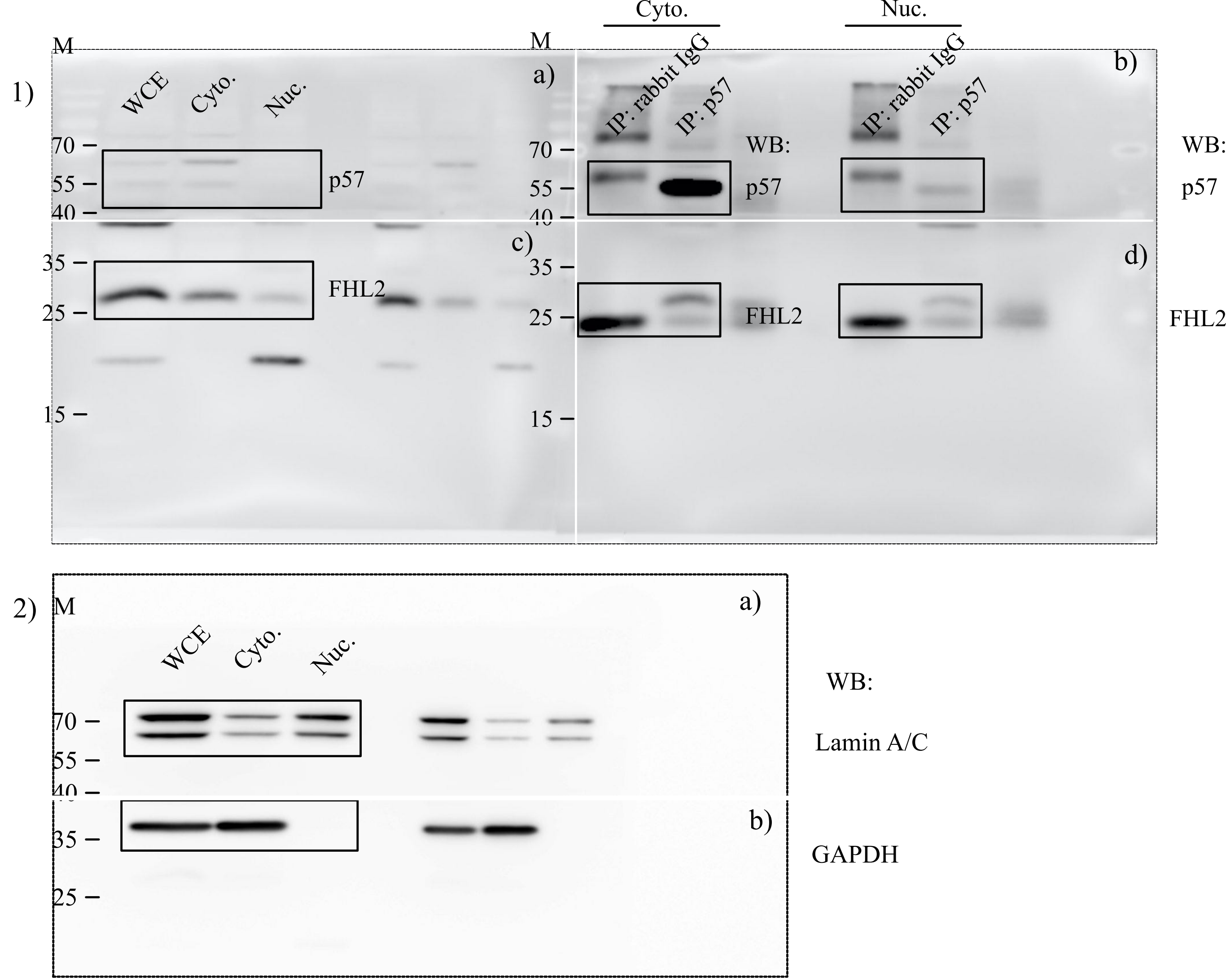

Supplementary Figure S4: Las4000 generated TIFF images from immunoblots, approximate cropped areas are indicated by rectangles and were used to assemble Fig. 3c in the main article. Image 1 represents four PVDF-membranes (a-d), a and b were probed for p57 and used to show input p57 expression (a) and IPed p57 (b). Membranes c and d were probed for FHL2 and used to show input FHL2 expression (c) and co-IPed FHL2 (d). Image 2 represents two PVDF-membranes, a was probed for Lamin A/C and b for GAPDH. Approximate boundaries of membranes are indicated by white thick vertical and/or horizontal lines. Boundaries of the TIFF-images are marked by black dashed rectangles.

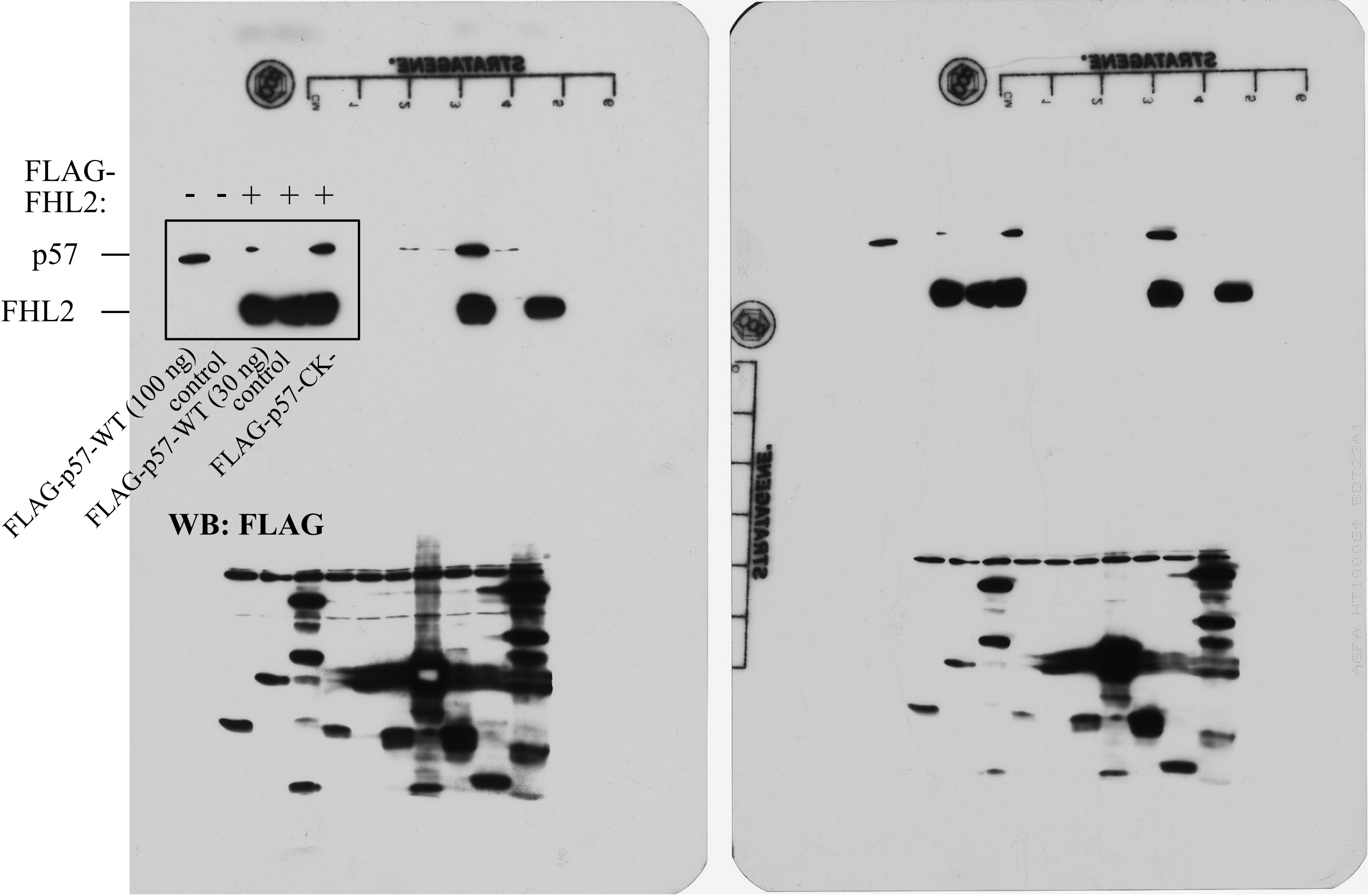

Supplementary Figure S5: original scans from immunoblots, approximate cropped areas are indicated by rectangles. Processed for Fig. 4b in article.

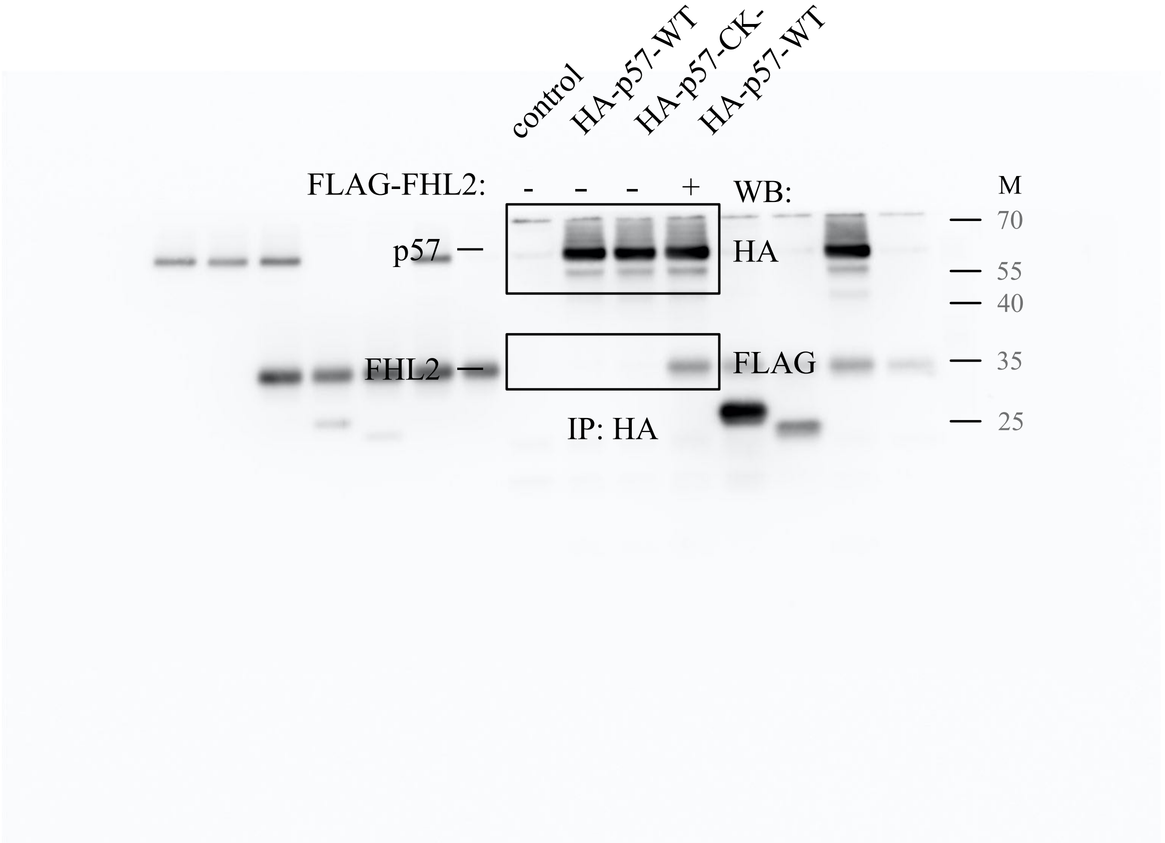

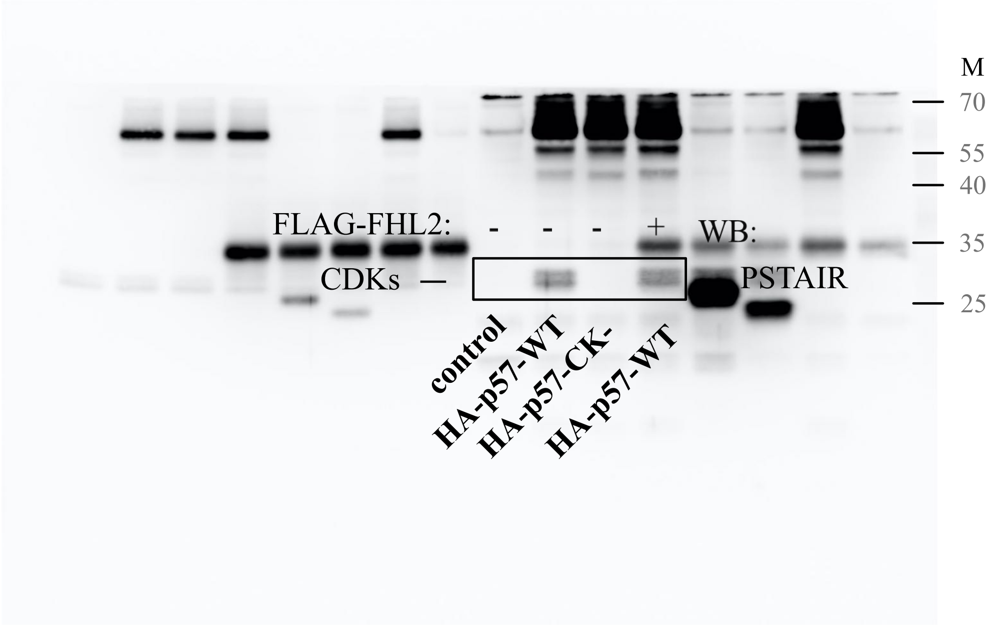

Supplementary Figure S6: Las4000 generated TIFF images from immunoblots, approximate cropped areas are indicated by rectangles. Processed for Fig. 4c in article.

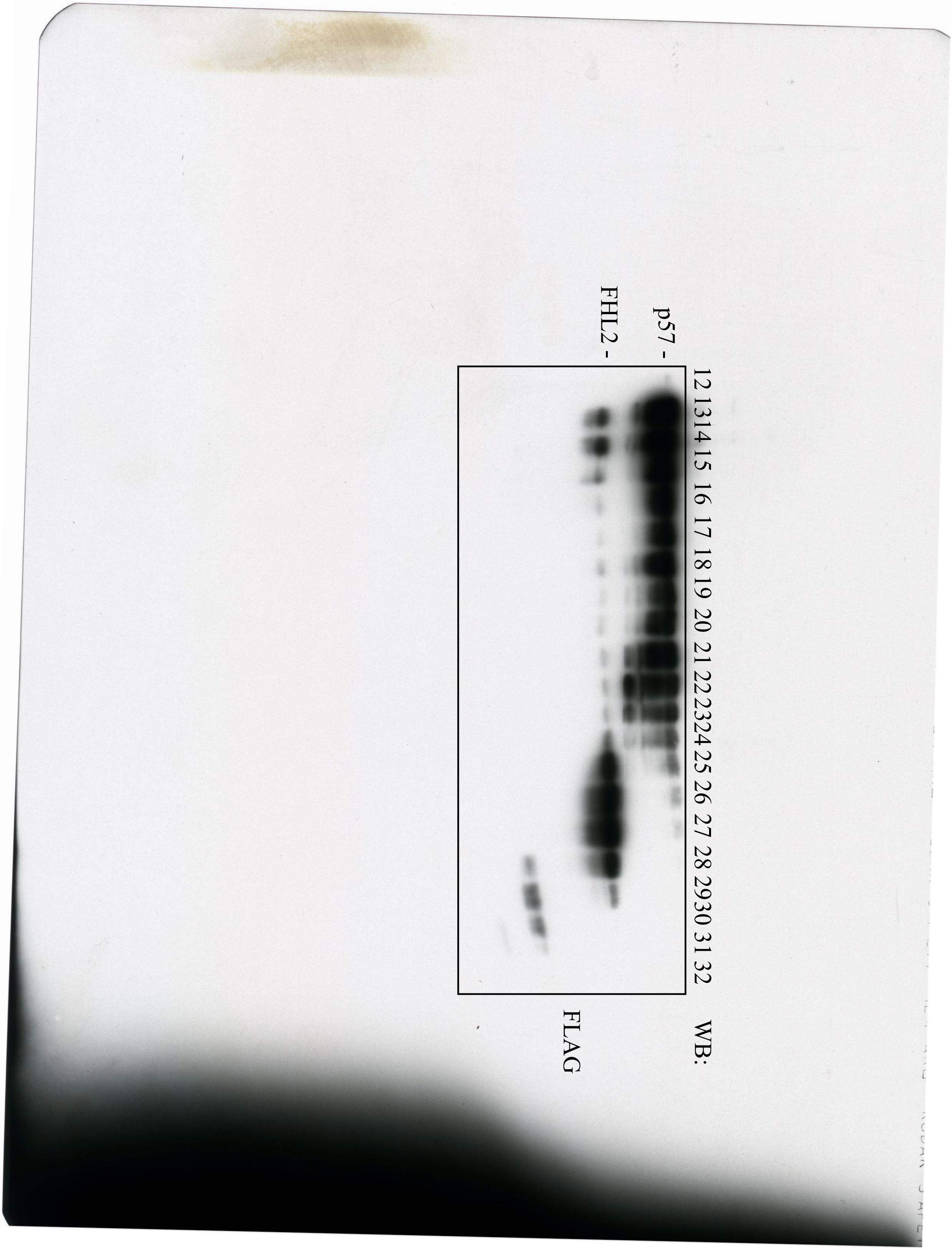

Supplementary Figure S7: original scans from immunoblots, approximate cropped areas are indicated by rectangles. Processed for Fig. 7c in article.

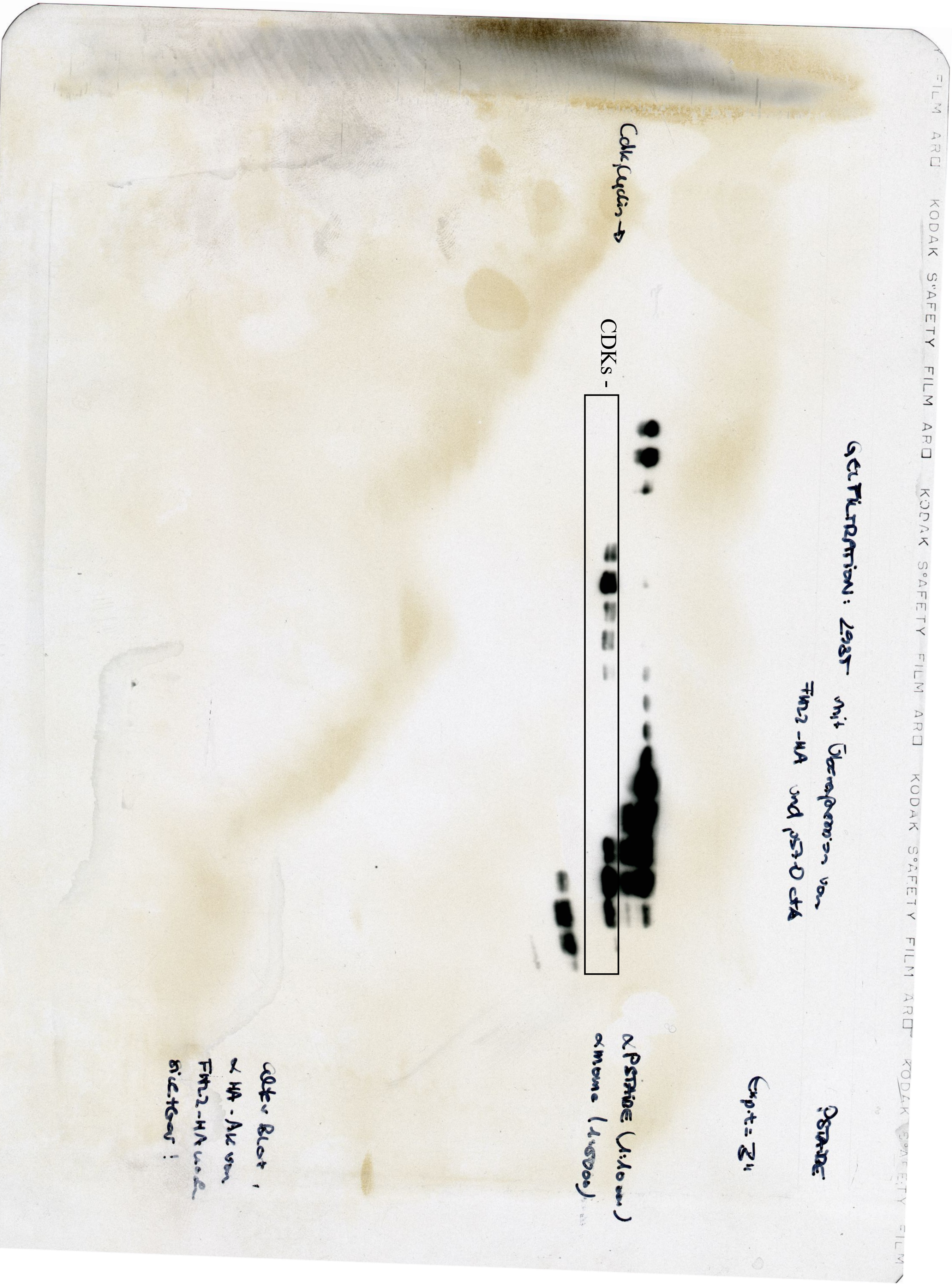

Supplementary Figure S7 (continued): original scans from immunoblots, approximate cropped areas are indicated by rectangles. Processed for Fig. 7c in article.

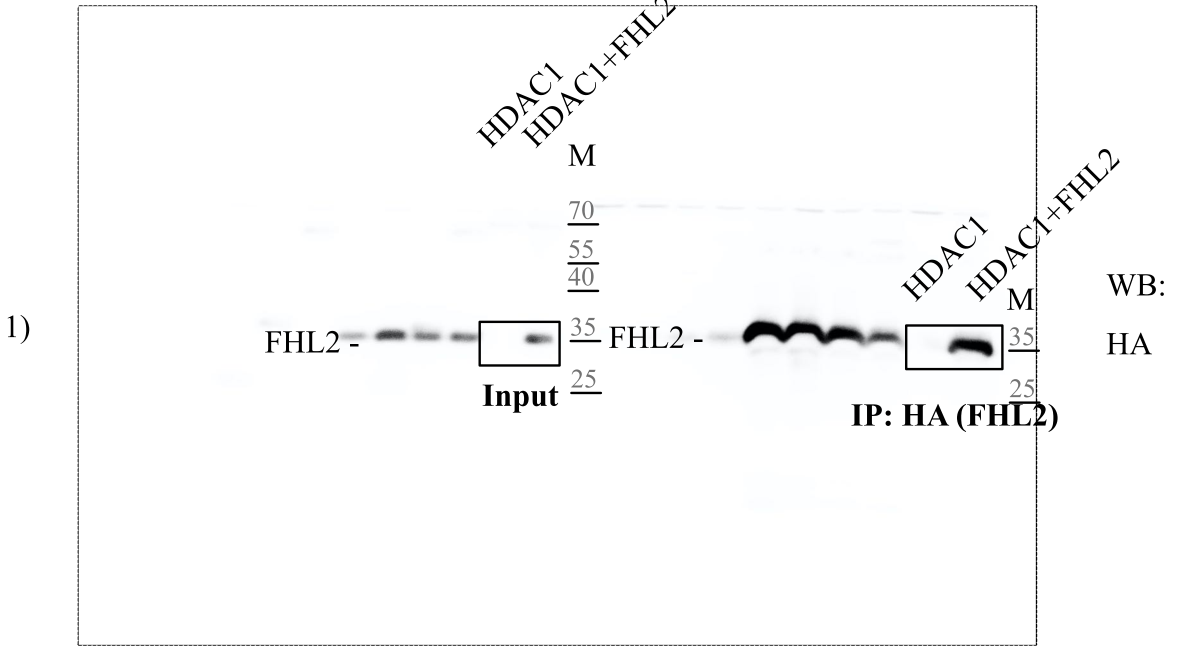

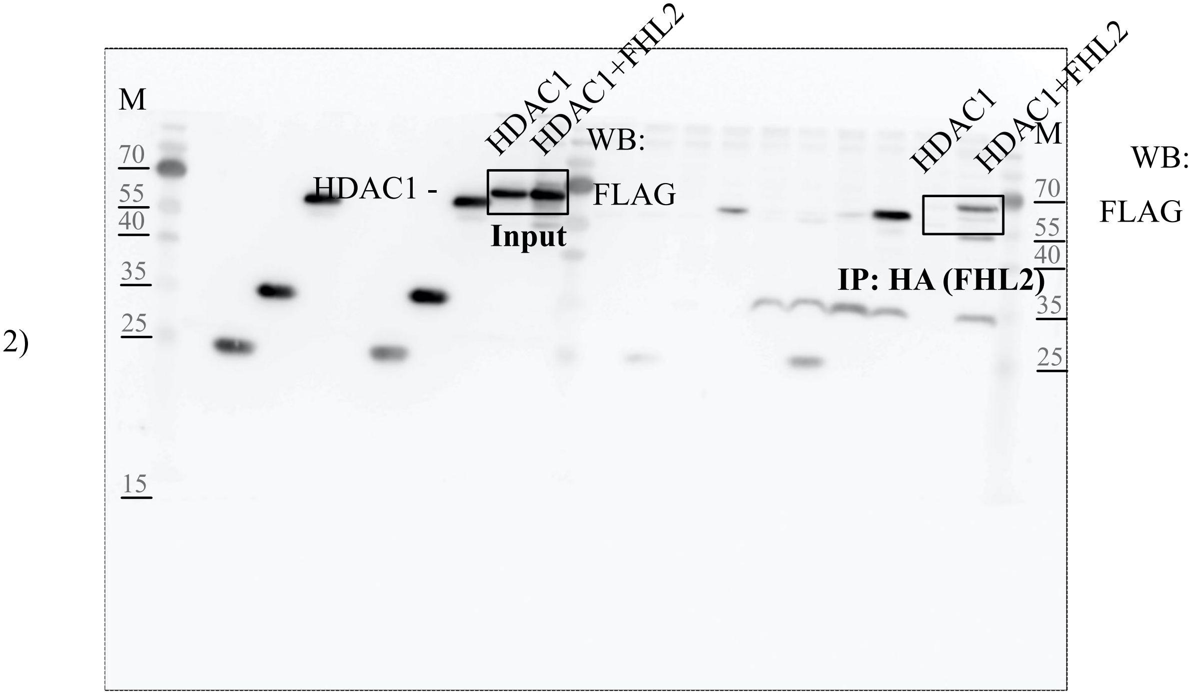

Supplementary Figure S8: Las4000 generated TIFF images from immunoblots, approximate cropped areas are indicated by rectangles and were used to assemble Fig. 8a in the main article. Upper image (1) was generated after probing with HA-antibody, the lower image (2) after reprobing the same PVDF-membrane with FLAG-antibody. Boundaries of the TIFF-images are marked by black dashed rectangles.

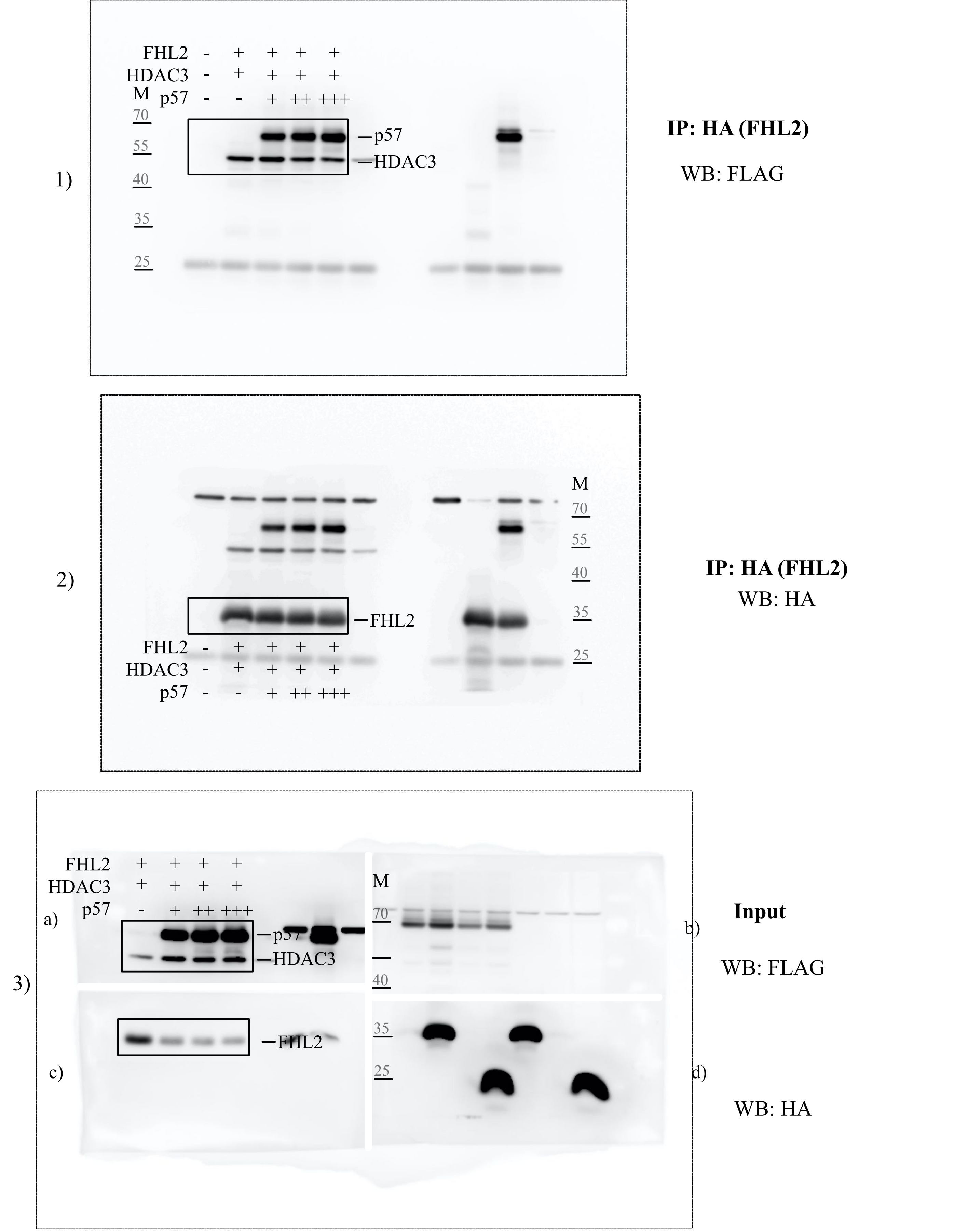

Supplementary Figure S9: Las4000 generated TIFF images from immunoblots, approximate cropped areas are indicated by rectangles and were used to assemble Fig. 8c in the main article. Upper image (1) was generated after probing with FLAG-antibody, image 2 after reprobing the same PVDF-membrane with HA-antibody. In image 3 PVDF-membranes a and c are relevant and show input levels for p57 and HDAC3 after probing with FLAG-antibody (a) and for FHL2 after probing with HA-antibody (c). Approximate boundaries of membranes are indicated by white thick vertical and horizontal lines. Boundaries of the TIFF-images are marked by black dashed rectangles.

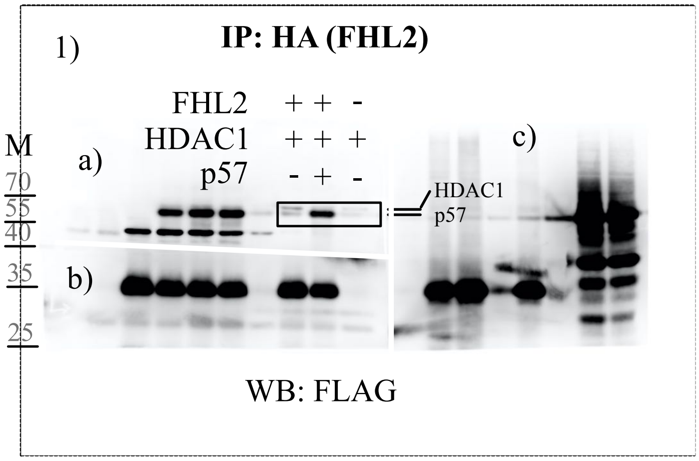

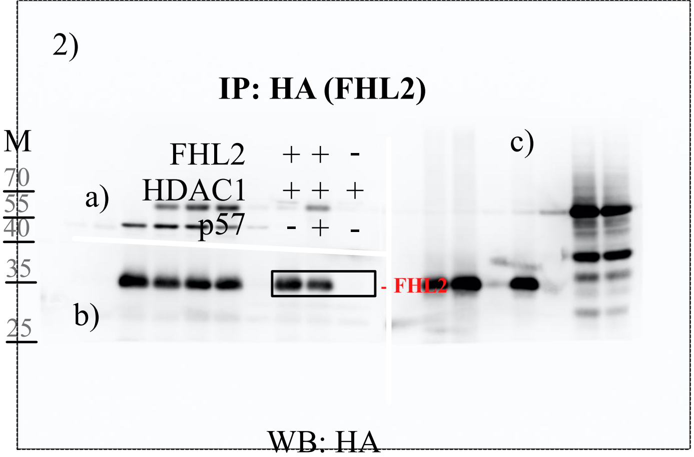

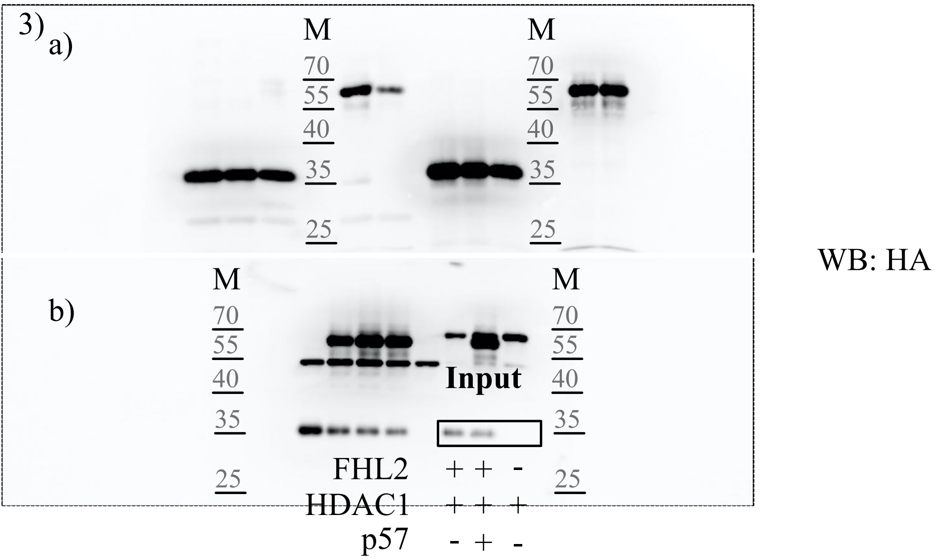

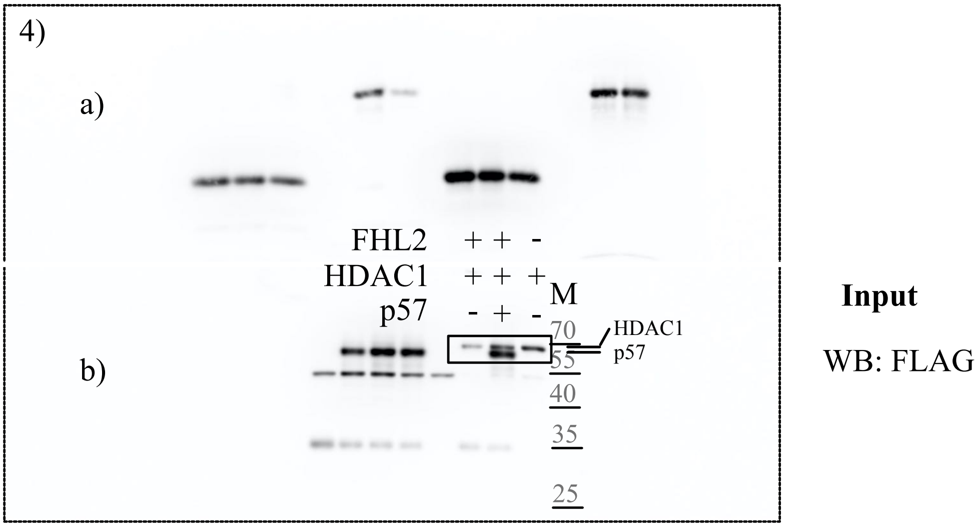

Supplementary Figure S10: Las4000 generated TIFF images from immunoblots, approximate cropped areas are indicated by rectangles and were used to assemble Fig. 8d in the main article. Numbers 1 to 4 represent individual images characters a and b individual PVDF-membranes (cutting indicated as white thick lanes). Images 1, 2 and 3, 4 differ only in their exposure times. Image 1 (membrane a) was used to detect co-IPed FLAG-HDAC1 and -p57 and image 2 (membrane b) to detect IPed HA-FHL2. Image 3 (membrane b) was used to show input HA-FHL2-levels and image 4 (membrane b) input FLAG-HDAC1 and -p57. White thick vertical and/or horizontal lines indicate more than one PVDF-membrane per imaging. Boundaries of the TIFF-images are marked by black dashed rectangles.

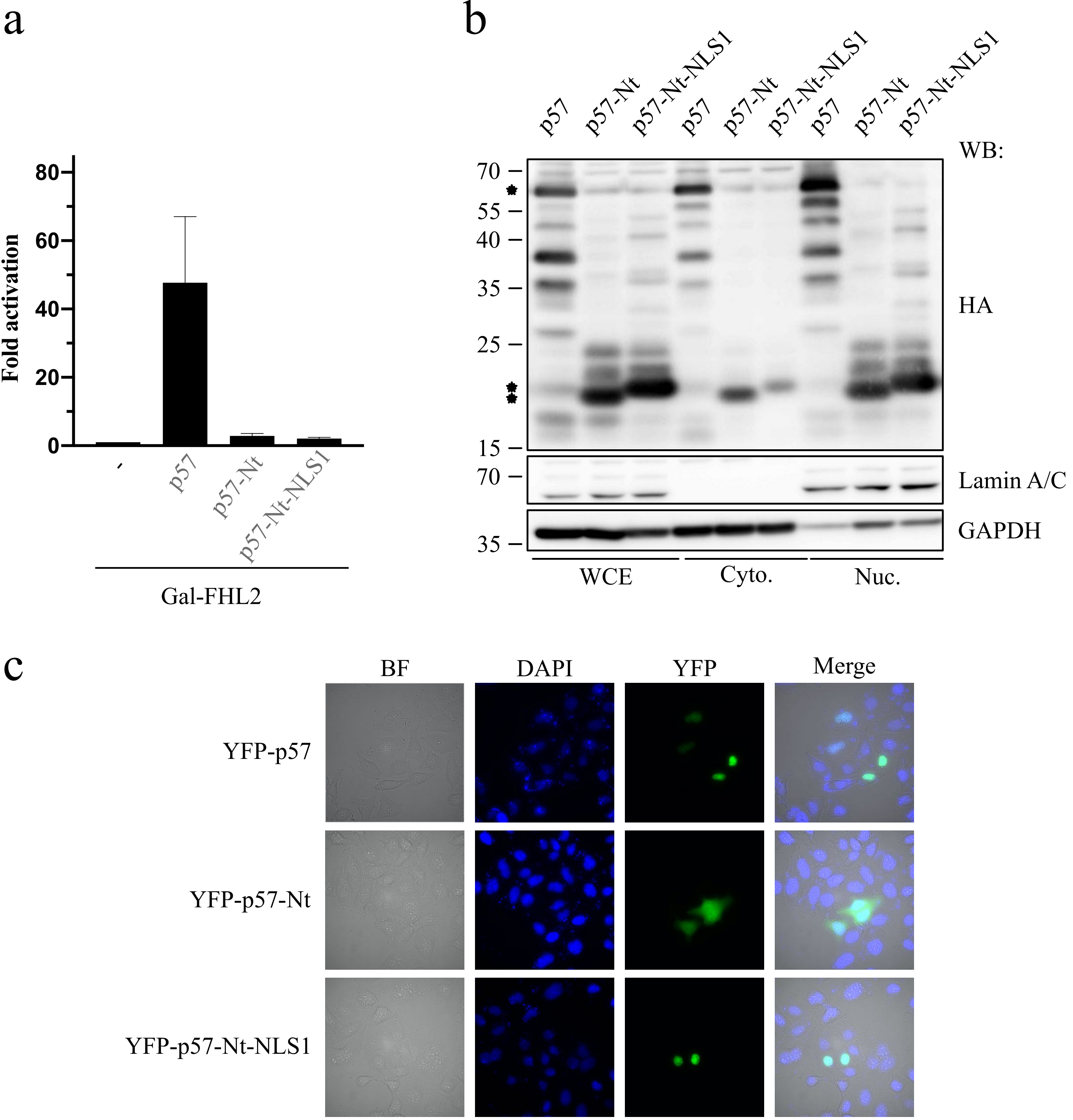

Supplementary Figure S11: Increased nuclear localisation of the p57 aminoterminus does not induce Gal-FHL2 activity. **(a)** 293FR cells were transfected with Gal-FHL2 alone or in combination with plasmids coding for p57 or the indicated mutants. As an internal control for transfection efficiencies a Renilla luciferase coding plasmid was included in all transfections. Renilla normalised firefly values of Gal-FHL2 transfected cells were set to one and values from p57 co-transfected cells are expressed relative to Gal-FHL2. Data are shown as the mean of four independent experiments, standard deviation is included as error bars. **(b)** Increased nuclear localisation of p57-Nt-NLS1 mutant. Subcellular fractionation and immunoblot analysis from transfected 293 cells using an HA-specific antibody (upper panel). Expressed proteins and respective subcellular fractions are indicated above and below the panels (WCE = whole cell extract, Cyto. = cytoplasmic fraction, Nuc. = nuclear fraction). *: HA-signal corresponding to p57 and mutants. Expression of Lamin A/C controlled for the quality of cytoplasmic and GAPDH for nuclear fraction. (c) p57-Nt-NLS1 localises mainly to the nucleus. Analysis p57 and aminoterminal p57 mutants subcellular localization in living cells. YFP-p57, YFP-p57-Nt and YFP-p57-Nt-NLS1were overexpressed in HeLa cells. Proteins were detected by excitation of the fused fluorophores. Bright field (BF), DNA was stained with DAPI.

d

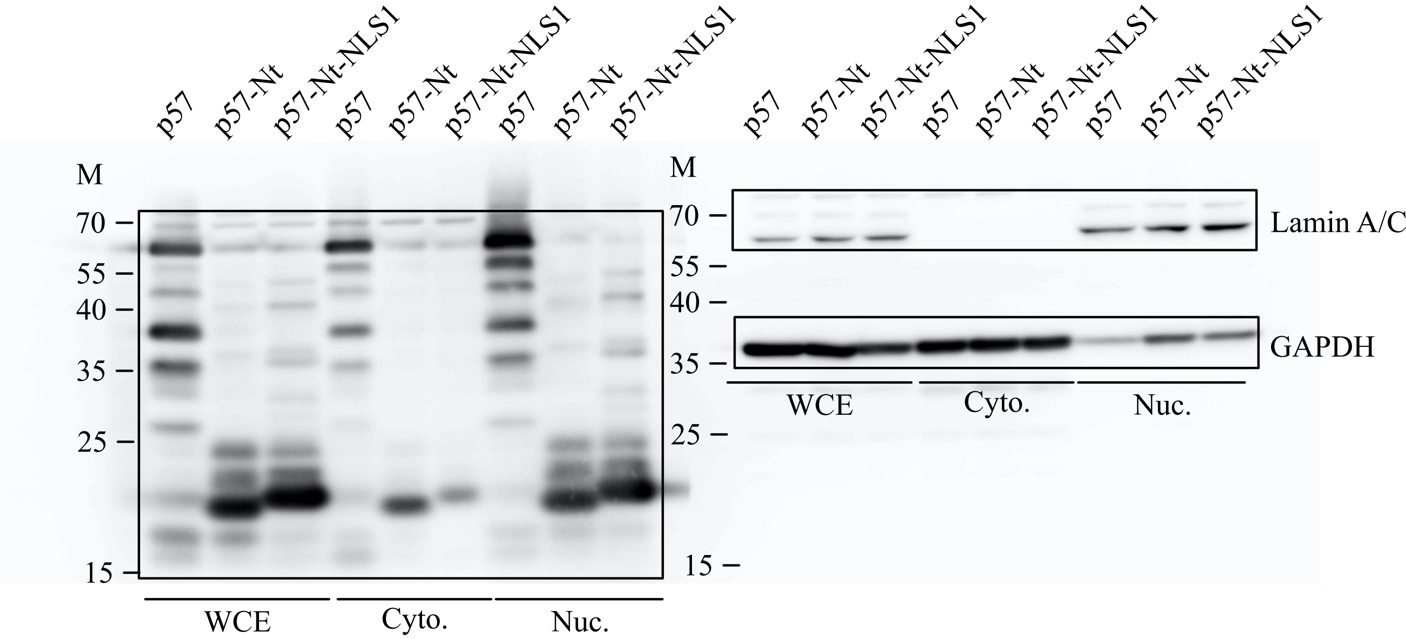

Supplementary Figure S11 (continued): (d) Las4000 generated TIFF images from immunoblots, approximate cropped areas are indicated by rectangles. Processed for supplementary Fig. S11b.
